# Gelatin-Based Multifunctional Hydrogels for Sports Injury Repair: Musculoskeletal and Nervous System Perspectives

**DOI:** 10.3390/gels12060493

**Published:** 2026-06-02

**Authors:** Jiangmei Cao, Yutong Wang, Hongchao Zhang, Yanan Lu, Jie Wu, Haihua Li, Wenyan Wang, Xu Duan, Xing Gao

**Affiliations:** 1 School of Kinesiology and Health, Graduate School, Harbin Sport University, Harbin 150008, China; cjm19885090620@163.com (J.C.); wangyutong@hrbipe.edu.cn (Y.W.); a13351455315@163.com (H.Z.); 15689732682@163.com (W.W.); duanxu1627@163.com (X.D.); 2 Inner Mongolia Academy of Agricultural and Animal Husbandry Science, Hohhot 010031, China; 3 College of Art and Physical Education, Kyungil University, Gyeongsan-si 38428, Republic of Korea; wujie@hrbipe.edu.cn; 4 Guangxi Key Laboratory of Tumor Immunology and Microenvironmental Regulation, Guilin Medical University, Guilin 541000, China; 18300964948@163.com

**Keywords:** gelatin, natural biomaterials, multifunctional hydrogels, sports injuries, tissue engineering, regenerative rehabilitation

## Abstract

Sports injuries, especially musculoskeletal and neurological types from strenuous exercise, are a global public health challenge. Characterized by a high incidence and slow recovery, these injuries differ from typical trauma, often resulting in severe mechanical transmission loss and an imbalanced immune microenvironment. Consequently, standard interventions struggle to achieve true tissue regeneration. Gelatin, a collagen-derived biomaterial, offers RGD-mediated cell adhesion, MMP-responsive degradation, and high modifiability. These qualities make it an excellent foundation for biomimetic repair scaffolds. This paper reviews the design principles and recent advances in gelatin-based multifunctional hydrogels in sports medicine. First, we analyse their structure and engineering advantages. Next, we summarise strategies and mechanisms for modules like conductivity, antibacterial activity, self-healing, stimulus responsiveness, and tissue adhesion. The review links these modules to types of injuries: bone or cartilage, tendon or ligament, skeletal muscle, spinal cord, and peripheral nerve. It clarifies their clinical and translational value in remodelling immune microenvironments, regulating electrophysiology, promoting interfacial regeneration, and restoring motor function. This review provides focused insights from materials science and sports rehabilitation to advance precision treatments for sports injuries.

## 1. Introduction

Sports injuries are a growing global health concern. They have a high incidence and slow recovery. Increased public health awareness and competitive sport participation raise injury rates in the musculoskeletal (articular cartilage, tendons, ligaments, bones) and nervous systems (spinal cord, peripheral nerves) [1]. Unlike general trauma or chronic degenerative diseases often discussed in conventional tissue engineering, sports injuries are distinct due to their high-energy impact and repetitive cyclic loading. They severely compromise biomechanical transmission, reduce tissue load-bearing capacity, and disrupt the immune microenvironment. These factors make recovery challenging. For instance, extreme pivot shifts and shear forces frequently cause anterior cruciate ligament (ACL) ruptures and meniscus tears, whereas sudden, explosive accelerations often trigger Achilles tendon ruptures and severe muscle strains. These specific biomechanical failures require repair matrices that not only provide structural bridging but also withstand dynamic, repetitive stretching and loading without early degradation. Conventional wound dressings or rigid scaffolds fail under such demanding cyclic motion, whereas gelatin-based networks, particularly those engineered with dynamic cross-linking, exhibit exceptional viscoelasticity and fatigue resistance, making them uniquely suited for this mechanically dynamic rehabilitation environment [2,3,4,5]. Traditional methods, such as suturing or grafting, restore anatomy but rarely reverse pathology or enable true regeneration. They also face donor shortages and additional trauma [6]. New therapies urgently need biomimetic scaffolds that regulate regeneration and combine mechanical with biological functions [7].

Among biomaterials, hydrogels stand out for their three-dimensional network and extracellular matrix (ECM)-like properties. Specifically, those made from naturally sourced gelatin, a collagen derivative, have advantages over synthetic polymers and other natural polysaccharides [7,8]. Gelatin retains collagen’s RGD (arginine–glycine–aspartic acid) sequence, which promotes cell adhesion. This provides natural biochemical targets for stem cell recognition, adhesion, and proliferation. Furthermore, gelatin-based hydrogels retain matrix metalloproteinase (MMP)-sensitive degradation sites, enabling enzyme-mediated breakdown as new tissue forms. This unique feature means scaffold degradation can synchronise with tissue remodelling. Additionally, gelatin’s side chains (-NH_2_ and -COOH) allow for versatile ‘functional programming’ of the material. Consequently, gelatin-based hydrogels are well-suited to address the multifaceted challenges of sports injury repair [9,10].

Thanks to these structural properties, gelatin-based hydrogels have excellent biocompatibility and very low immunogenicity. This gives a solid biological foundation for tissue engineering [11]. However, single-component gelatin struggles in the complex pathological microenvironment of sports injuries. These conditions increase oxidative stress, bacterial infection, and disrupt electrical signal transmission [12]. As a result, research has shifted from focusing only on biocompatibility to designing multifunctional materials that interact with their environment. Using strategies like physical blending, chemical grafting, and nanocomposites, researchers have added conductivity, antibacterial and antioxidant properties, and smart responsiveness to gelatin networks [13,14,15,16]. This creates a smart hydrogel platform with sensing, response, and repair functions. Tendon repair presents another challenge. Natural tendons lack the capacity to transmit bioelectric signals. This impedes real-time rehabilitation monitoring. To address this, Li et al. [17] introduced a smart artificial tendon. They integrated the conductive polymer PEDOT:PSS into the hydrogel matrix. This approach yields a conductive gelatin hydrogel with excellent biocompatibility. It supports motor function recovery and real-time monitoring of stress in in vitro joint models. The technology provides a data-driven solution for preventing tendon re-rupture. In bone injury repair, sports-related fractures often cause ongoing aseptic inflammation. They also trigger bursts of reactive oxygen species (ROS), which block callus formation. Huang et al. [18] developed a ROS-responsive gelatin-based hydrogel loaded with dimethyl fumarate to counteract this. This system uses a microenvironment-responsive mechanism to release drugs as needed, based on local ROS levels. Higher ROS levels lead to greater release of anti-inflammatory drugs. This boosts anti-inflammatory and antioxidant effects, restoring an immune microenvironment that supports bone regeneration. Open sports injuries present hard-to-heal wounds and frequent antibiotic resistance. These conditions challenge normal infection management. Researchers [19] have engineered a bacterium-responsive, self-activating antimicrobial hydrogel built from composite chitosan (CS) and gelatin. The material is sensitive to pH changes caused by bacterial metabolism. It activates antimicrobial activity only when needed. It eradicates bacterial biofilms and releases functional ions like Cu^2+^ to foster angiogenesis. This offers an innovative solution for complex, infected sports injuries.

These developments show that gelatin-based multifunctional hydrogels have become therapeutic platforms for tissue regeneration, not just simple cell scaffolds. While numerous existing reviews have extensively covered the fundamental synthesis of gelatin and gelatin methacryloyl (GelMA) hydrogels, or their broad applications in general tissue engineering [20,21], they generally treat tissue defects as static environments. Few studies systematically correlate the design strategies of multifunctional gelatin materials with the unique, highly dynamic, and biomechanically demanding microenvironments typical of sports injuries. The unique contribution of this review lies in its deeply interdisciplinary perspective, which bridges materials science and sports medicine. Specifically, we explicitly map advanced hydrogel functionalities—including dynamic self-healing for cyclic mechanical loads, electroactivity for neuromuscular signal restoration, and stimuli-responsiveness for acute inflammatory bursts—to the distinct pathological requirements of bone, cartilage, tendon, muscle, and nerve repair following sports-related trauma. In this context, our review summarises recent research on gelatin-based multifunctional hydrogels for the repair of sports injuries. First, we analyse the molecular structure of gelatin and its role in sports medicine. Next, we discuss approaches to building multifunctional modules with properties such as conductivity, antibacterial activity, self-healing, smart responsiveness, and tissue adhesion. We then consider their applications in sports medicine and explore their use in repairing bone/cartilage, tendons/ligaments, muscles, and nerves. Finally, we highlight the paradigm-shifting potential of emerging technologies in this field, such as artificial intelligence (AI)-assisted biomaterial design, 4D bioprinting, bioelectronic interfaces, exosome-loaded hydrogels, CRISPR-engineered cell–hydrogel systems, and smart rehabilitation-integrated biomaterials. To ensure these cutting-edge concepts can successfully transition into clinical practice, this review goes beyond merely celebrating preclinical triumphs. Instead, we critically address the formidable translational barriers—including GMP-compliant manufacturing, sterilization techniques, and regulatory approval pathways. By confronting these clinical realities, we aim to provide a pragmatic roadmap for bridging the gap between lab-scale engineering and successful commercialization.

## 2. Gelatin for the Repair of Sports Injuries

As a hydrolysed derivative of collagen, gelatin carries the main biological features of the ECM. Its controllable physicochemical properties—like solubility, gel strength, and environmental responsiveness—make it a promising biomaterial for clinical use in sports injury repair [22]. While synthetic polymers serve as viable alternatives, gelatin exhibits superior cell recognition, immunotolerance, and controllable degradation. These benefits have been shown in many experimental studies [23]. To elucidate why gelatin aligns so perfectly with the stringent requirements of sports injury repair, the following sections examine its core molecular mechanisms, biological properties, and material advantages.

### 2.1. Active Tripeptide Repeat Sequences: Receptor Binding and Cellular Regulatory Targets

The biological functions of gelatin derive from its specific amino acid composition. Its primary structure is rich in glycine (Gly), proline (Pro) and hydroxyproline (Hyp); this characteristic Gly-Pro-Hyp tripeptide repeat sequence forms the basis for maintaining the rigidity of the gelatin molecular chain and the formation of local triple-helix structures, directly determining the mechanical stability and thermoreversible gelation behaviour of the hydrogel. More crucially, gelatin retains the key RGD cell adhesion sequence found in collagen. This sequence acts as a molecular key, specifically recognising and binding to integrin receptors on the cell surface, thereby activating the intracellular focal adhesion kinase signalling pathway, which governs cell adhesion, spreading, and differentiation [24]. This mechanism is particularly important in bone and cartilage repair. For example, the work of Lin et al. [25] provides strong evidence of this. When constructing a composite scaffold based on collagen (COL) and carboxymethyl chitosan (CMC), they found that the unmodified scaffold exhibited weak affinity for bone marrow mesenchymal stem cells (BMSCs), with the cells appearing round and poorly spread out; however, upon the chemical introduction of additional RGD peptides, the adhesion rate of BMSCs surged to over 80% within 24 h, with the cells exhibiting a healthy spindle-shaped morphology and forming stable cell clusters. This comparative experiment not only confirmed the pivotal role of the RGD sequence but also indirectly demonstrated the inherent advantage of gelatin, as a naturally RGD-rich material, in promoting stem cell engraftment following sports injuries.

### 2.2. Excellent Biocompatibility and Low Immunogenicity

As a naturally sourced protein, gelatin undergoes enzymatic hydrolysis to remove the terminal peptide regions of collagen that are primarily responsible for immunogenicity, thereby exhibiting extremely low antigenicity [26]. For sports medicine repair materials intended for long-term implantation (such as artificial ligaments or cartilage scaffolds), the host immune response triggered by the material is a key factor determining success or failure. The in vivo safety assessment conducted by Chauhan et al. [27] is highly representative. They implanted gelatin hydrogels into the subcutaneous tissue of rats and carried out continuous observation for up to 30 days. Histological section results revealed no significant inflammatory cell infiltration around the implantation site, nor were typical foreign body reactions (FBR), such as thickening of the fibrous capsule, observed. This excellent in vivo biocompatibility enables gelatin-based hydrogels to act as an invisible scaffold, providing a gentle microenvironment for the regeneration of damaged tissue without triggering an excessive inflammatory storm. However, a critical limitation of current literature is that long-term biosafety data are overwhelmingly derived from small-animal models, which fail to replicate the complex human immune microenvironment. To cross the translational threshold, rigorous validation in large-animal models (e.g., pigs or sheep) under cyclic physiological loading is urgently required to exclude chronic foreign body responses.

### 2.3. Enzyme-Responsive Smart Degradation Mechanism

Unlike synthetic polymers (such as PLA and PCL), which rely primarily on non-specific hydrolysis, gelatin degradation exhibits significant microenvironment dependence [28]. The gelatin molecular chain framework naturally retains specific cleavage sequences for MMPs, particularly MMP-2 and MMP-9. During the repair of sports injuries, damaged tissue secretes MMPs to remodel the matrix, causing the gelatin scaffold to undergo responsive degradation, thereby achieving perfect synchronisation between scaffold degradation and the ingrowth of new tissue. Wang et al. [29] ingeniously exploited this property to design an MMP-responsive gelatin-based drug delivery system for the repair of infected wounds. By regulating the cross-linking density of the gelatin, they ensured that the curcumin release rate was positively correlated with MMP concentration at the wound site (i.e., the level of inflammation). Experimental results demonstrated that this on-demand release strategy achieved higher healing rates than conventional dressings in a rat full-thickness skin defect model. This proves that gelatin is not merely a passive filler, but rather a stimulus-responsive active matrix capable of feedback interaction with the pathological microenvironment. Furthermore, precisely tuning the degradation kinetics of gelatin hydrogels is critical for achieving optimal biomechanical integration in sports medicine. Since sports-related tissues experience substantial mechanical loading, premature scaffold degradation can lead to structural collapse and repair failure. Conversely, a well-controlled degradation profile enables a progressive transfer of physiological loads from the degrading hydrogel matrix to the newly forming ECM. This dynamic mechanical matching not only prevents stress shielding but also provides continuous mechanotransduction cues to endogenous cells, thereby effectively promoting functional tissue remodeling under physical stress [30].

### 2.4. Functionalisation via Reactive Side Chains and Potential for Multi-Step Processing

The abundance of reactive functional groups (amino, carboxyl and hydroxyl groups) on the side chains of gelatin makes it a highly versatile material platform. Through simple chemical modifications (such as methacrylation, resulting in GelMA), gelatin can be transformed from a thermosensitive material into a photopolymerisable material, thereby enabling its use in advanced manufacturing processes such as 3D printing. For example, in the field of bone repair, Yu et al. [31] utilised GelMA in combination with strontium–calcium silicate to prepare a bio-ink, successfully 3D-printing a bone repair scaffold with a precisely biomimetic microporous structure. This scaffold not only offers tunable mechanical strength but also demonstrates exceptional osteogenic induction capacity in a rat critical-size cranial defect model. In the field of neural repair, gelatin’s excellent miscibility makes it easy to combine with conductive materials. Han et al. [32] prepared a nerve conduit that combines electrical conductivity with biocompatibility by compositing the conductive polymer PEDOT with a gelatin/chitosan matrix. In a rat sciatic nerve defect model, this conduit not only physically bridged the severed ends but also effectively transmitted electrical signals, significantly reducing disuse atrophy in the distal muscles. In the field of anti-infection, gelatin networks also serve as ideal carriers for nanoparticles. Research by Hia et al. [33] confirmed that, following the in situ loading of zinc oxide nanoparticles (ZnO) or lysozyme onto gelatin/chitosan networks, the material exhibited significant antibacterial zones against both Gram-positive and Gram-negative bacteria, whilst effectively disrupting bacterial membrane structures.

In summary, gelatin perfectly meets the multifaceted requirements for materials in sports injury repair, thanks to its RGD-mediated adhesion activity, MMP-responsive degradation properties, proven in vivo safety, and strong engineering potential. Although most commercially available gelatin currently derives from mammals, fish-derived gelatin—as an alternative source with similar functionality but a lower melting point—is gradually coming to the fore through methods such as cross-linking modification. This development addresses concerns regarding religious dietary restrictions (such as halal and kosher) and the risk of zoonotic diseases, thereby offering more diverse options for sports rehabilitation across different populations worldwide [34]. Nevertheless, the practical scalability of animal-derived gelatin is severely hindered by batch-to-batch variability in molecular weight and impurity profiles, which directly undermines product reproducibility. Furthermore, establishing compatible sterilization methods remains a persistent challenge; standard autoclaving or gamma irradiation frequently causes premature backbone cleavage, severely compromising the hydrogel’s initial mechanical integrity and long-term storage stability.

To clarify the clinical selection criteria for sports injury repair, Table 1 compares gelatin with other foundational biomaterials. Unlike synthetic polymers that lack cell-signaling moieties [28] or natural polysaccharides with uncontrollable degradation, gelatin uniquely bridges the biological–mechanical gap. By retaining native cell-recognition motifs and MMP-responsive cleavage sites, it provides an optimal template perfectly synchronized with tissue remodeling [7,8].

## 3. Design Strategies and Performance Realisation of Gelatin-Based Multifunctional Hydrogels

### 3.1. Gelatin-Based Conductive Hydrogels

In the repair of sports injuries, bioelectric signals play a crucial role in muscle contraction, nerve conduction and bone remodelling, whilst electrical stimulation (ES) has been shown to effectively promote cellular metabolism, proliferation and differentiation, thereby accelerating tissue regeneration [39]. However, conventional ES devices are typically made of rigid materials, making it difficult to achieve mechanical compatibility and stable coupling with soft, dynamic sports tissues, which limits their application in the repair of sports injuries [40]. Conductive hydrogels (CHs), as soft materials combining a highly hydrated, hydrophilic network with efficient electrical signal pathways, can both mimic the mechanical flexibility and physiological microenvironment of natural tissues and enable uniform electrical signal transmission, thereby effectively addressing the shortcomings of traditional ES devices. Gelatin, as a natural polymeric matrix, not only possesses excellent biocompatibility and cell-adhesion properties but also provides active groups that serve as a scaffold for the construction of conductive networks, making gelatin-based CHs an ideal carrier for linking exogenous electrical stimulation with endogenous bioelectric regulation in the repair of sports injuries.

**Figure 1:**
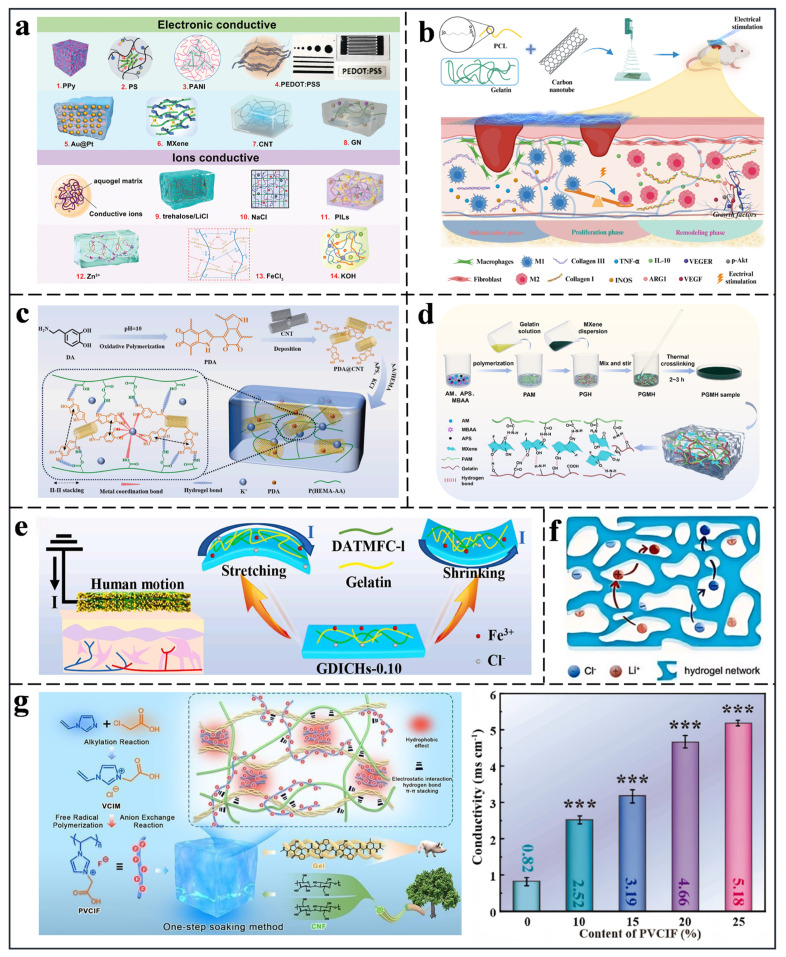
(**a**) Examples of common conductive matrices and their applications [41]. (**b**) An electroactive scaffold composed of PCL/GE/CNT, fabricated via electrospinning and combined with electrical stimulation to promote skin repair [45]. (**c**) Surface engineering of carbon nanotubes with polydopamine (PDA) and the mechanism by which it enhances interfacial compatibility [46]. (**d**) Polymerization mechanism of MXene-reinforced PAM/Gel conductive hydrogels [50]. (**e**) High-sensitivity sensing mechanism of alternating dense/porous ionic hydrogel matrices [54]. (**f**) Conduction and temperature-responsive adhesion mechanism of LiCl-doped hydrogels [55]. (**g**) Microscopic cross-linking mechanisms and conductivity variations in PVCIF-enhanced ionic hydrogels at different concentrations, *** *p* < 0.001 vs. the 0% PVCIF group [56].

The most common method for preparing CHs involves directly doping the hydrogel matrix with conductive components. The electrochemical and mechanical properties of CHs can be tuned by altering the conductive fillers, dopants or hydration state. Currently, based on differences in the conductive mechanism of hydrogels, CHs can be broadly classified into electronic CHs, ionic CHs and mixed ionic–electronic CHs. While the structural design (Figure 1a) and performance characteristics of different conductive hydrogels (CHs) dictate their suitability for specific injuries, a critical comparison reveals distinct trade-offs. Electronic CHs typically offer superior and stable electrical conductivity but struggle with biodegradability and potential long-term cytotoxicity. Conversely, ionic CHs present excellent biocompatibility and tissue-like flexibility, yet their signal transmission stability often degrades under the cyclic mechanical loading typical in sports rehabilitation [41].

#### 3.1.1. Gelatin-Based Electronic Hydrogels

Electronic hydrogels derive their conductivity from the directed migration of free electrons within conductive fillers, such as carbon nanomaterials [42]. While these nanomaterials share common advantages like high specific surface areas and continuous conjugated π-bond networks [43], their integration into gelatin matrices requires distinct engineering strategies to overcome specific material limitations.

For instance, carbon nanotubes (CNTs) significantly enhance matrix conductivity, yet their inherent hydrophobicity severely limits aqueous dispersion [44]. To address this, Chen et al. [45] developed electrospun PCL/gelatin/CNT scaffolds that achieved conductivities matching normal skin (1 × 10^−4^ to 0.26 S/m), providing excellent bioelectrical compatibility for tissue stimulation, as shown in Figure 1b. As illustrated in Figure 1c, Wang et al. [46] applied a polydopamine (PDA) coating to CNTs, introducing oxygen-containing functional groups that facilitated uniform dispersion via hydrogen bonding and π-π stacking with the hydrogel matrix.

Compared to the dispersion challenges of CNTs, graphene oxide (GO) naturally possesses abundant hydroxyl and carboxyl groups, enabling more straightforward interfacial interactions with gelatin. Wang et al. [47] demonstrated that blending GO with a PLA/gelatin matrix significantly improved mechanical performance—yielding a tensile strength of 2.4 MPa—while simultaneously reducing the water contact angle and conferring intrinsic antibacterial activity. This makes GO composites highly suitable for multifunctional wound dressings.

However, neither CNTs nor GO can match the metallic-level electrical conductivity offered by MXene nanosheets, which utilize highly mobile, delocalized interlayer electrons [48,49]. As illustrated in Figure 1d, by firmly anchoring MXene within a polyacrylamide-gelatin double-network via electrostatic interactions and hydrogen bonds, Ma et al. [50] effectively suppressed nanosheet aggregation. This unique design yielded a remarkable electrical conductivity of 0.076 S/m and sustained stable signal output even under severe deformation. Further studies have successfully employed dynamic covalent bonds to address MXene’s susceptibility to environmental oxidation, ensuring the long-term stability of the composite hydrogels [51,52].

#### 3.1.2. Gelatin-Based Ionic Hydrogels

In contrast to electronic hydrogels, ionic hydrogels rely on the migration of free ions (e.g., metal salts, ionic liquids) within a hydrated polymer network [53]. While generally exhibiting lower absolute conductivity than electronic counterparts, ionic hydrogels offer superior transparency, anti-freezing properties, and mechanical flexibility that more closely mimic human soft tissues.

To balance the inherent trade-off between mechanical robustness and ionic mobility, Fu et al. [54] designed a gelatin-based hydrogel (GDICHs) featuring an alternating dense/porous network, as shown in Figure 1e. The dense regions provided mechanical load-bearing capacity, while the porous channels facilitated the efficient migration of Fe^3+^ and Cl^−^ ions, enabling ultra-high sensing sensitivity for sports rehabilitation monitoring. Furthermore, ionic systems excel in providing stimuli-responsive bio-interfaces. As illustrated in Figure 1f, Li et al. [55] developed an adhesive ionic hydrogel utilizing LiCl that responds to body temperature. It firmly adheres to the skin during exercise to ensure stable electromyographic signal acquisition and can be painlessly removed upon cooling, effectively addressing the dynamic monitoring needs of sports injuries.

To mitigate the risk of free-ion leakage associated with simple metal salts, polyelectrolytes serve as a stable alternative. As depicted in Figure 1g, Song et al. [56] employed a polyionic liquid (PVCIF) to induce gelatin aggregation via the salting-out effect. This non-covalent interaction strategy not only reinforced the mechanical network but also achieved a notable conductivity of 5.18 mS·cm^−1^, demonstrating a highly controllable approach to fabricating robust ionic conductors.

#### 3.1.3. Gelatin-Based Composite Conductive Hydrogels

Electrical conductivity typically depends on the conductive polymers that form a conductive network within the hydrogel; however, ionic conductivity arises from a polymer network permeated by a wealth of free ions, including salts and amphoteric ionic compounds [41]. Hybrid ion–electron-conductive hydrogels (CH) achieve synergistic conduction of electrons and ions by simultaneously constructing electronic conduction pathways (e.g., conductive filler networks) and ionic conduction pathways (e.g., ion source channels) within a gelatin matrix; their overall performance is superior to that of CHs based on a single conductive mechanism [57]. For example, Yin et al. [58] synthesised a series of gelatin-based, multifunctional, recyclable and biodegradable hybrid conductive hydrogels using CNTs, cellulose sol and sodium citrate (Na_3_Cit) as additives. Thanks to the electronic conductive network formed by CNTs and the ionic conductive pathways provided by Na_3_Cit, this hydrogel achieved a conductivity of up to 0.93 S/m. Ion exchange between PEDOT:PSS and metal halides drives phase separation within the hydrogel, thereby enabling ultra-high conductivity. In a similar study, Montazerian et al. [59] used sulphated alginate (AlgS) as a hydrophilic dopant for PEDOT to replace conventional PSS, significantly improving the dispersibility (5-fold) and electrical conductivity (20-fold) of PEDOT-based hydrogels. Furthermore, this modification imparts biodegradability, enhances biocompatibility, and reduces fibrous encapsulation, successfully overcoming the limitations of conventional PSS. Beyond establishing an efficient electron-transport pathway, the critical biomedical significance of these conductive networks lies in their capacity to mediate stem cell interactions through bioelectrical signals. When applied to injury sites under dynamic movement, these hydrogels act as an artificial electroactive microenvironment that can interface directly with endogenous stem cells. By translating mechanical deformation or microcurrent stimulation into intracellular electrical signals, the conductive matrix triggers the opening of voltage-gated ion channels, subsequently activating downstream signaling pathways (such as MAPK/ERK or PI3K/Akt) to direct the lineage-specific differentiation of bone marrow mesenchymal stem cells (BMSCs) or neural stem cells (NSCs), thereby significantly accelerating musculoskeletal and neural regeneration [60]. Despite the enhanced performance of composite CHs, several critical limitations hinder their clinical applicability. First, the biodegradation kinetics of the conductive network often mismatch the rapid tissue remodeling rate, potentially leaving unresolved fragments that trigger chronic foreign body responses. Furthermore, while their immunomodulatory potential under electrical stimulation is promising, large-scale manufacturing (scalability) remains challenging due to the complex multi-step crosslinking required to prevent filler agglomeration. Future research must prioritize inherently conductive and biodegradable polymers to successfully decouple electrical performance from long-term cytotoxicity.

### 3.2. Gelatin-Based Antimicrobial Hydrogels

While many biomaterial studies focus on generic wound healing, effective infection control in sports medicine presents unique, highly demanding scenarios. Acute trauma in extreme sports frequently results in heavily contaminated open fractures or severe turf abrasions, exposing deep tissues to diverse environmental pathogens. Furthermore, the extensive use of surgical hardware—such as interference screws for ACL reconstruction or suture anchors for rotator cuff repair—drastically elevates the risk of implant-associated infections and devastating post-operative complications [61]. In these scenarios, once bacterial biofilms form on implants or avascular sports tissues, they trigger intractable chronic inflammation that conventional systemic antibiotics fail to penetrate. Consequently, localized, highly efficient antimicrobial interventions—such as functionalized gelatin hydrogels—are imperative to salvage the graft and preserve the athlete’s career [62]. Gelatin-based hydrogels are regarded as excellent matrix materials for constructing ideal antimicrobial platforms due to their inherent biodegradability, cell-adhesion properties, and ease of functionalisation. Depending on their antimicrobial mechanisms, antimicrobial hydrogels can be divided into two main categories: intrinsically antimicrobial hydrogels and antimicrobial agent-releasing hydrogels. The former rely on cationic groups or active structures within their own composition to disrupt the integrity of bacterial cell membranes through physical contact, thereby achieving a bactericidal effect; the latter act as carriers for antimicrobial agents, delivering drugs locally at the site of injury to achieve effective bactericidal concentrations in the infected microenvironment, thereby delivering highly efficient and rapid antimicrobial effects [63].

#### 3.2.1. Gelatin-Based Intrinsically Antimicrobial Hydrogels

Intrinsically antimicrobial hydrogels can be formulated using natural or synthetic polymers (such as CS, QCS, and ε-PL), as well as by incorporating antimicrobial small molecules or ionic liquids (ILs). These materials exhibit good biocompatibility and degradability. The functional groups within this structure confer antimicrobial activity to the natural polymers and enhance the antimicrobial performance and manufacturability of the hydrogels [63].

**Figure 2:**
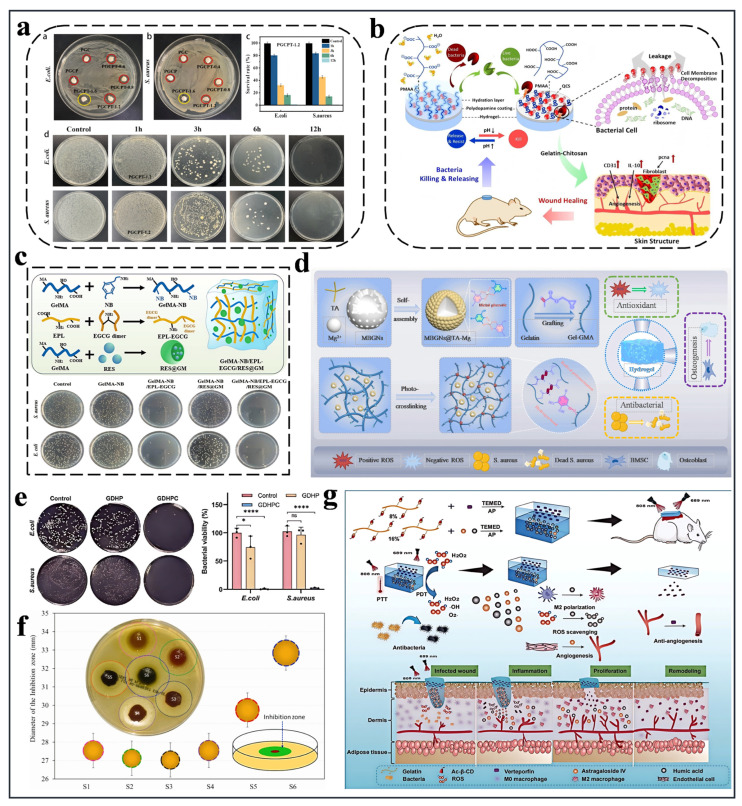
(**a**) In vitro antibacterial zones and quantitative cell survival rates of triple-network PVA/CS/Gel hydrogels against *E. coli* and *S. aureus* [65]. (**b**) Schematic mechanism of a pH-responsive, switchable antimicrobial and anti-fouling hydrogel [66]. (**c**) Gelatin-based composite hydrogels covalently grafted with ε-PL and natural plant polyphenols, and their synergistic antibacterial mechanism [69]. (**d**) Construction strategy and synergistic antibacterial mechanisms of Gel-GMA hydrogels loaded with TA-Mg^2+^ metal–phenolic networks [70]. (**e**) Quantitative surface antibacterial activity and bacterial viability analysis of GDHPC hydrogels, * *p* < 0.05; **** *p* < 0.0001; ns, no significant difference [75]. (**f**) Inhibition zone assays evaluating the antibacterial efficacy of CuNP-loaded multi-material hydrogels [81]. (**g**) Schematic illustration of bilayer HAs-AS@Vt@BGACD hydrogels for synergistic PTT/PDT antibacterial therapy [86].

CS is a natural cationic polymer prepared by deacetylation of chitin. It possesses inherent antimicrobial properties and is readily modifiable; the amino groups on CS are easily protonated, conferring inherent bactericidal activity to the polymer. The antimicrobial activity of CS can be enhanced through quaternisation or by grafting hydrophobic alkyl groups onto the amino groups [63,64]. Based on this, Zhang et al. [65] developed a triple-network hydrogel comprising polyvinyl alcohol (PVA)/CS/gelatin, which was modified with tannic acid (TA) and 3-carboxyphenylboronic acid (3-CPBA). In this study, CS was chemically modified with 3-CPBA, and a stable triple-network structure was formed by cross-linking TA via boronate bonds. Thanks to the incorporation of CS and TA, the PGCPT hydrogel exhibited 100% antibacterial activity against both *Escherichia coli* and *Staphylococcus aureus* within 12 h (Figure 2a), providing an important reference for antibacterial hydrogels used in skin wound repair. Fang et al. [66] successfully developed a smart hydrogel dressing based on chitosan (CS) and gelatin. By covalently grafting quaternary chitosan (QCS) and pH-responsive polymethylacrylate (PMAA) onto the hydrogel surface via PDA, they achieved a cyclable antimicrobial function comprising ‘resistance–bactericidal action–release’, as illustrated in Figure 2b. This hydrogel ingeniously exploits the acid-producing nature of bacterial metabolism: at physiological pH, it relies on a hydrated layer of PMAA to resist initial bacterial adhesion; whereas in the acidic microenvironment caused by infection, the PMAA chains undergo conformational collapse, exposing the underlying QCS to perform contact-mediated bactericidal action; following bactericidal action, dead bacteria automatically detach, and the material surface subsequently returns to its anti-adhesive state. ε-PL is a natural antimicrobial peptide derived from Streptomyces albus, consisting of 25–30 lysine residues. Owing to its high density of positive charges, it can disrupt the negatively charged bacterial cell membrane through electrostatic interactions, exhibiting potent, broad-spectrum bactericidal activity against both Gram-positive and Gram-negative bacteria whilst resisting the development of resistance [67]. Based on this, Guo et al. [68] developed a dual-network hydrogel based on PVA and gelatin. By grafting ε-PL onto gelatin and combining it with PDA and serotonin hydrochloride, they successfully endowed the hydrogel with excellent tissue adhesion and antibacterial properties. To further enhance the antibacterial efficacy of natural peptides, Cao et al. [69] proposed a strategy in another study involving the covalent grafting of ε-PL onto natural plant polyphenols (EGCG) and incorporating this into a GelMA network to construct a multifunctional composite hydrogel (Figure 2c). Experimental results indicate that, owing to the contact-penetration mechanism of ε-PL and the synergistic antimicrobial activity of polyphenols, this hydrogel exhibits antimicrobial efficiencies of up to 92.2% and 97.4% against *Staphylococcus aureus* and *Escherichia coli*, respectively, without the need for external physical stimulation. Furthermore, it possesses strong haemostatic and anti-inflammatory properties, demonstrating significant potential in infection control and emergency wound healing. Similar to EGCG, TA, another highly representative natural plant polyphenol and organic acid, not only forms a dense network of hydrogen bonds within hydrogels but also inherently possesses excellent properties, such as antioxidant, antibacterial, and haemostatic effects. As shown in Figure 2d, Deng et al. [70] developed a gelatin-based hydrogel (GM/MBTM) containing TA, composed of glycidyl methacrylate-modified gelatin (Gel-GMA) and mesoporous bioglass (MBGNs). In this system, the cationic modified gelatin network can initially disrupt the negatively charged bacterial cell membranes through electrostatic interactions; simultaneously, as a natural polyphenolic compound, TA’s phenolic hydroxyl groups can further interfere with microbial enzyme activity and metabolic processes. The metal–phenol network formed by TA and Mg^2+^ synergistically enhances this dual-action bactericidal effect. Compared with the unmodified control group and the group containing only MBGNs, the GM/MBTM hydrogel containing the TA-Mg^2+^ network exhibited the strongest antibacterial effect against *Staphylococcus aureus*, with a bactericidal efficiency exceeding 80%. Furthermore, inspired by the natural compound CS, researchers have developed ILs composed of organic cations and anions with melting points below 100 °C. ILs typically disrupt bacterial membranes by creating pores, leading to rapid cell leakage and death. In the work by Romero-Antolín et al. [71], a dual-action hydrogel comprising GelMA as the matrix and a polyionic liquid (PIL) with salicylate (Sal) anions was proposed to achieve both anti-inflammatory and antibacterial activity. The Sal-modified PIL confers antibacterial and anti-inflammatory properties to the hydrogel. Compared with the control hydrogel without PIL, the PIL hydrogel containing Sal anions achieved a bacterial kill rate of nearly 100% within 4 to 8 h. Notably, the PIL hydrogel containing bromide anions but no salicylate also exhibited potent antibacterial activity, suggesting that the antibacterial effect primarily stems from the cationic portion of the IL. This work successfully integrates imidazolyl-PIL, which acts via membrane-disruption, into the gelatin hydrogel network, endowing the material with potent, rapid, broad-spectrum antibacterial activity without significantly altering the hydrogel’s physicochemical properties. This provides a solid experimental basis and theoretical support for the design of novel IL-based antibacterial hydrogel dressings.

#### 3.2.2. Gelatin-Based Antimicrobial-Releasing Hydrogels

For hydrogels loaded with antimicrobial agents, antibiotics (such as ciprofloxacin, vancomycin and gentamicin) are undoubtedly low-cost and effective options. Antibiotics are secondary metabolites produced by microorganisms or higher plants and animals; they effectively kill or inhibit microorganisms through specific mechanisms such as inhibiting bacterial cell wall synthesis, increasing cell membrane permeability and interfering with protein synthesis [63]. The three-dimensional porous structure of hydrogels enables them to serve as delivery systems, allowing lower doses of antibiotics to be delivered directly to the site of infection and achieving high local concentrations [72]. This reduces the incidence of antibiotic misuse, improves antibiotic utilisation, and offers good biocompatibility [64]. Ciprofloxacin (CIP) is a fluoroquinolone antibiotic with broad-spectrum antibacterial activity, used to treat bacterial infections, including skin and bone infections. The antibacterial mechanism of CIP involves inhibiting bacterial DNA synthesis and gyrase, leading to bacterial death. Liu et al. [73] successfully prepared a GelMA/carboxymethylated sulphated xanthan gum composite hydrogel loaded with CIP. This composite hydrogel, which possesses biomimetic mineralisation capabilities, effectively inhibits bacterial growth through the controlled release of CIP hydrochloride (over 24 h). Furthermore, this system significantly enhances local mechanical properties (with the shear modulus increasing to 13.9 kPa, 2.6 times higher than the original hydrogel) and maintains excellent biocompatibility (cell viability > 99.5% at 72 h), ultimately facilitating the regeneration of infected bone defects. Beyond pathogen eradication, these antimicrobial hydrogels play a pivotal role in active immune modulation. In sports injuries, following bacterial clearance, accumulated debris and excessive ROS often sustain a chronic inflammatory microenvironment. Multifunctional gelatin hydrogels address this by serving as an immunomodulatory switch; by scavenging ROS or capturing pro-inflammatory cytokines, they drive the phenotypic transition of local macrophages from the pro-inflammatory M1 to the pro-healing M2 phenotype. This M2 polarization suppresses the inflammatory cascade and initiates the secretion of regenerative growth factors, transitioning the wound into a constructive tissue-remodeling phase [74]. Tang et al. [75] developed a multifunctional hydrogel based on the cross-linking of gelatin–dopamine with hyaluronic acid–phenylboronic acid, loaded with CIP hydrochloride (CIP-H), to create a wound microenvironment-responsive GDHPC hydrogel. As shown in Figure 2e, this hydrogel exhibited an antibacterial rate of nearly 100% against both *Escherichia coli* and *Staphylococcus aureus*. Furthermore, in infection models, the GDHPC hydrogel effectively cleared bacteria from the wound, significantly reducing colony formation. Although there are significant concerns regarding bacterial resistance and nephrotoxicity, vancomycin remains a clinically important therapeutic agent [76]. Vancomycin is a glycopeptide antibiotic with potent antibacterial activity against Gram-positive bacteria. Its mechanism of action involves inhibiting a key step in bacterial cell wall synthesis, thereby preventing peptidoglycan cross-linking and leading to bacterial lysis and death. Lin et al. [77] prepared a biodegradable gelatin–silica hybrid loaded with vancomycin. The antibacterial activity of the hydrogel against *Staphylococcus aureus* and *Escherichia coli* was evaluated using zone-of-inhibition assays. The results indicated that the hydrogel effectively inhibited bacterial growth, and that vancomycin was released continuously as the carrier degraded uniformly, avoiding the ‘burst release’ issue associated with conventional antimicrobial carriers. This sustained, localized release profile perfectly aligns with the stringent clinical requirements for preventing and treating severe implant-associated infections in sports orthopaedics, such as deep-seated infections following meniscal repair or hardware colonization in bone fracture fixation. By maintaining effective antimicrobial concentrations within the local microenvironment for up to 5 months without burst-induced cytotoxicity, this hydrogel platform offers a robust strategy to safeguard post-operative sports rehabilitation.

Although antibiotics have achieved considerable success in antimicrobial therapy over the past few decades, the adverse consequences of their overuse have posed a serious challenge to the medical field, leading to the active development of antibiotic-free treatment strategies. Compared with antibiotics, metal ions and metal oxides can enhance and sustain antimicrobial activity, whilst bioextracts and antimicrobial polymers exhibit good antimicrobial activity and biocompatibility. Generally speaking, these alternatives can replace antibiotics, thereby reducing the incidence of antibiotic abuse and the development of resistance. Metal ions, such as Ag^+^, Cu^2+^, Zn^2+^ and Au^+^, possess broad-spectrum antimicrobial activity and are widely used in antimicrobial materials. Among these, silver nanoparticles (AgNPs) are widely utilised in antimicrobial applications due to their excellent antimicrobial properties [64]. Zhang et al. [78] successfully constructed an AgNPs@CQ composite system by double-coating AgNPs with carboxymethylcellulose (CMCD) and quaternised cellulose (QCD), and incorporated it into a gelatin-based hydrogel. The incorporation of AgNPs@CQ into the hydrogel endowed it with excellent antibacterial properties, significantly inhibiting bacterial growth. The double-layer coating structure effectively delayed the release of Ag^+^, prolonging the antibacterial activity. Furthermore, Wang et al. [79] prepared a gelatin–agar composite hydrogel containing AgNPs synthesised using carbon dots as a reducing agent, which can be used to treat infected wounds. The results indicated that this composite hydrogel exhibited a bacterial inhibition rate exceeding 99% against *Staphylococcus aureus* and *Escherichia coli*. The composite hydrogel can also be used as a wound dressing to promote the healing of infected wounds in mice. Wu et al. [80] synthesised AgNPs via a TA-based green synthesis method and, using GelMA and HA as carriers, prepared injectable dual-network composite hydrogels via photocrosslinking, which exhibited immediate bactericidal effects against *Staphylococcus aureus* and *Escherichia coli*. Furthermore, CuNPs have also attracted widespread attention from researchers. Krishna et al. [81] developed a 3D-printable composite hydrogel based on gelatin/PVA/xanthan gum, into which CuNPs were incorporated via glycolaldehyde cross-linking. This hydrogel exhibited good biocompatibility. Its antibacterial performance was evaluated using the zone of inhibition method; as shown in Figure 2f, the CuNP-containing hydrogel demonstrated significant antibacterial activity against *E. coli*. Furthermore, the incorporation of CuNPs markedly increased the compressive modulus of the hydrogel (reaching 4.18 MPa) and its overall compressive strength (up to 690 kPa), enhancing its antibacterial activity against common pathogens whilst enabling controlled degradation. Owing to its excellent biocompatibility and enhanced mechanical properties, this multi-material hydrogel has emerged as a promising candidate for the development of customised, patient-specific tissue-engineering scaffolds. Compared with AgNPs, CuNPs offer advantages such as lower cost, easy in vivo release and significant antimicrobial properties. In addition to the aforementioned metal nanoparticles, AuNPs and ZnNPs also exhibit good antimicrobial activity.

However, unmodified or highly concentrated metal nanoparticles often exhibit poor biocompatibility and potential cytotoxicity, which can limit their broad biomedical applications. Against this backdrop, the introduction of novel antimicrobial strategies has significantly reduced the incidence of drug-resistant bacteria. Among these, non-antibiotic-dependent approaches such as photothermal therapy (PTT) and photodynamic therapy (PDT) have been widely adopted in antimicrobial research. PTT acts directly on bacteria by inducing thermal stress, disrupting the cell wall integrity of *Staphylococcus aureus* and *Escherichia coli*, resulting in wrinkling and rupture of the bacterial surface [82]. As a non-invasive therapy, PDT does not induce drug resistance. PDT relies on photosensitizers (PS) to generate ROS under light of an appropriate wavelength, thereby damaging bacterial cell membranes and inactivating DNA and proteins, thereby achieving sterilisation. However, due to their hydrophobic nature and poor water solubility, most PS require further research and resolution. With the increasing prevalence of drug-resistant bacteria, PDT, a broad-spectrum antimicrobial therapy, is emerging as a treatment strategy for bacterial infections [83,84]. Zhao and his team [85] developed a gelatin/alginate composite hydrogel doped with a cyanine derivative (Cn) for photodynamic antimicrobial applications. Upon illumination, the photosensitiser Cn is excited, converting oxygen molecules in the surrounding environment into ROS, particularly singlet oxygen. ROS oxidise and destroy key biological macromolecules such as bacterial cell membranes, proteins and nucleic acids, leading to structural damage and bacterial death. Among these, the C3 molecule with a short alkyl chain demonstrated the optimal antibacterial effect due to its superior spatial expansion structure and positive charge, which facilitates interaction with bacterial membranes. This work provides an effective solution for light-controlled wound healing against drug-resistant bacteria.

With the growing threat of drug-resistant bacteria, the controllability and other advantages of PDT and PTT have paved the way for new strategies to combat them. Compared with monotherapy, the combination of PTT and PDT yields more significant antibacterial effects. Furthermore, this approach can reduce the incidence of side effects, offering potential applications in the treatment of drug-resistant bacteria [64]. As shown in Figure 2g, Zha et al. [86] prepared a multifunctional bilayer nano-composite hydrogel based on gelatin/acryloyl-β-cyclodextrin, in which the lower layer was loaded with humic acid (HAs) and astragaloside IV (AS), whilst the upper layer was loaded with vitiporfin (Vt). Under dual irradiation with 808 nm and 689 nm lasers, the antibacterial effect was exerted through the combined action of HAs-induced PTT and Vt-induced PDT. PTT generates localised high temperatures to disrupt bacterial structures, whilst PDT produces ROS to oxidise bacterial biomolecules. Results indicate that after 5 min of combined irradiation, approximately 95% inhibition of MRSA was achieved, as evidenced by SEM images showing severe disruption of the bacterial membrane. Concurrently, in vivo experiments demonstrated that this hydrogel significantly promotes the healing of infected wounds and reduces scar formation. This indicates that the HAs-AS@Vt@BGACD hydrogel has broad application potential for the treatment of skin wounds and wound-healing involving drug-resistant bacterial infections.

### 3.3. Gelatin-Based Self-Healing Hydrogels

In the treatment of sports injuries, hydrogel dressings often fail due to repeated mechanical loading from joints and muscles. Gelatin, with its abundant reactive side chains, offers a natural advantage for constructing dynamic cross-linked networks; upon damage, the interpenetration of polymer segments and the dynamic reorganisation of reversible bonds enable autonomous repair at the interface, thereby effectively preventing secondary material failure and providing sustained support [87]. Currently, gelatin-based self-healing hydrogels primarily rely on two mechanisms: non-covalent interactions (which confer rapid room-temperature healing capabilities but are limited in strength) and dynamic covalent bonds (which provide excellent mechanical support) [88]. However, achieving a balance between “rapid healing efficiency” and “high mechanical robustness” remains a significant translational challenge. Non-covalent networks heal almost instantaneously but often lack the modulus required for load-bearing tissues, whereas dynamic covalent networks provide sufficient strength but require longer healing times. Consequently, designing dual-network systems that synergistically integrate these bonds—without compromising the scaffold’s porosity and cellular infiltration—is currently the most critical requirement in sports medicine. Crucially, the dynamic reconfiguration of these cross-linked networks endows the hydrogel with unique viscoelasticity and exceptional fatigue resistance, which are paramount for mechanical matching with native tissues. Because sports-related tissues constantly endure substantial mechanical loading during movement, this adaptive viscoelastic behavior allows the hydrogel to efficiently dissipate concentrated stress. Consequently, it avoids interfacial detachment or catastrophic failure under long-term cyclic motion, ensuring robust biomechanical integration between the implant and the dynamic host tissue [89]. The following sections will explore specific strategies for these two mechanisms.

#### 3.3.1. Non-Covalent Interactions

**Figure 3:**
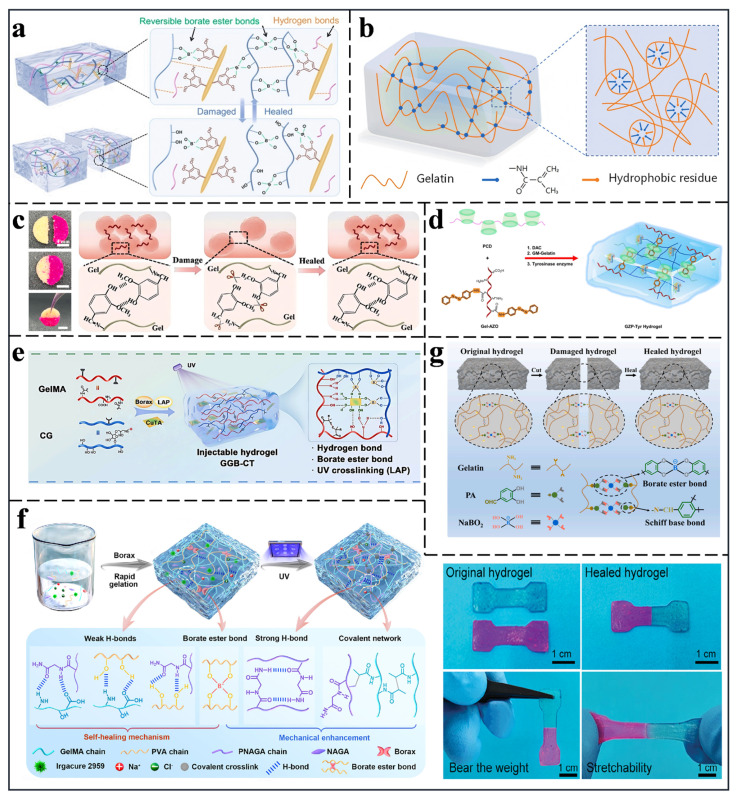
(**a**) Schematic illustration of the self-healing mechanism of PBGTC hydrogels [92]. (**b**) Schematic illustration of a hydrophobic association hydrogel based on GelMA [94]. (**c**) Illustration of the self-healing properties of VCM and a theoretical model of the self-healing mechanism [95]. (**d**) Schematic illustration of the preparation process for self-healing GZP-Tyr hydrogels [96]. (**e**) Schematic diagram of the synthesis of GGB-CT hydrogels [99]. (**f**) Schematic diagram of the preparation process, internal interactions and synthesis mechanism of GNPB hydrogels, as well as a demonstration of macroscopic mechanical self-healing [91]. (**g**) Schematic diagram of the self-healing mechanism of GPB hydrogels [103].

As a key non-covalent interaction, hydrogen bonding is one of the primary mechanisms responsible for the exceptional self-healing properties of gelatin-based hydrogels. Its mechanism essentially stems from the dynamic, reversible physical cross-linking between the abundant hydrogen bond donors and acceptors on gelatin molecular chains (such as the -C=O and -N-H groups on amide bonds and the -COOH and -OH groups on side chains). The advantage of this interaction lies in its highly dynamic reversibility: under external stress, the hydrogen bond network dissipates energy through preferential breaking, thereby preventing catastrophic failure of the material; once the stress is relieved, the broken hydrogen bonds rapidly reform under the drive of molecular thermal motion (chain diffusion), thus enabling autonomous healing of the damaged interface [90]. Compared to most covalent bonds, hydrogen bonds are relatively weak, which provides the basis for their rapid reversible breaking and reformation; however, the low bond energy (1–63 kJ·mol^−1^) also results in insufficient mechanical strength and poor stability in hydrogels, making them susceptible to interference from external mechanical forces or environmental factors (such as temperature, humidity and pH) [91]. To overcome this limitation, the development of multi-hydrogen-bonding systems is regarded as an effective approach to enhancing the mechanical properties of hydrogels. For example, Gu et al. [92] successfully prepared a strong and tough self-healing hydrogel based on multi-hydrogen bonding by compositing gelatin with PVA and TA-modified cellulose nanocrystals (TA@CNC). Studies have shown that gelatin, acting as a ‘bridge’, significantly increases the number of hydrogen bonds between PVA and TA@CNC, forming a high-density double hydrogen-bond network (Figure 3a). This structure not only effectively dissipates energy through reversible fracture, resulting in a hydrogel toughness as high as 246.09 kJ·m^−3^, but also achieves a self-healing efficiency of 93.3% within 120 s. This fully demonstrates that, through rational molecular design, a hydrogen-bond network can simultaneously endow materials with excellent mechanical properties and highly efficient self-healing capabilities.

In gelatin-based hydrogels, self-healing is achieved through a dynamic, reversible physical process involving hydrophobic interactions. Natural hydrophobic amino acid residues (such as valine and isoleucine) on gelatin molecular chains, or hydrophobic groups introduced through chemical modification, spontaneously aggregate in aqueous solution by ‘avoiding water molecules’ to form dynamic hydrophobic microdomains (micelles). These microdomains act as physical cross-linking points to construct a three-dimensional network. When damage is induced by an external force, the hydrophobic microdomains dissociate to dissipate energy, thereby preventing brittle fracture of the material; upon removal of the external force, the hydrophobic segments re-aggregate through molecular thermal motion, enabling network reconstruction and self-healing, whilst simultaneously conferring mechanical toughness and environmental stability to the hydrogel. It is worth noting that the formation and stability of such hydrophobic microdomains can be achieved through chemical modification or by adjusting the gelatin concentration [93]. In their study, Liao et al. [94] employed a chemical modification strategy to introduce methacryloyl groups into gelatin molecules, thereby significantly enhancing their hydrophobicity and promoting the aggregation of hydrophobic regions. This resulted in the formation of a physical network with micelles acting as cross-linking nodes; moreover, the micelle diameter increased markedly with increasing degree of methacryloyl substitution, indicating that more hydrophobic groups were aggregating (Figure 3b). This hydrogel exhibits excellent self-healing properties, rapidly restoring structural integrity after cutting and achieving multiple recoveries of modulus during cyclic strain testing (with the modulus immediately recovering to about 78% of its initial value upon strain removal), thereby validating its dynamic reversibility based on hydrophobic interactions. Inspired by the self-assembly behaviour of fire ants, Zhang et al. [95] developed vanillin-functionalised gelatin microspheres. Through the synergistic action of hydrophobic interactions and hydrogen bonding, they achieved autonomous aggregation of the microspheres and the construction of a macroscopic scaffold. This system completed structural reconstruction within 8 min of being cut and maintained morphological stability after injection, demonstrating its adaptability and self-healing potential in complex defect environments (Figure 3c).

Gelatin is rich in functional groups such as amino and carboxyl groups; achieving self-healing properties through host–guest molecular modification represents an effective strategy for constructing dynamic self-healing gelatin hydrogels. This strategy is based on specific recognition and reversible molecular-level binding between macrocyclic host molecules (such as cyclodextrins and cuparene) and complementary guest molecules. This interaction combines high selectivity with dynamic reversibility; although its binding strength is weaker than that of covalent bonds, it is stronger than many other non-covalent bonds, thereby enabling the formation of robust and durable physical cross-linking points within the hydrogel network. When the hydrogel is disrupted by external forces, these host–guest bonds can undergo reversible dissociation and reconformation, effectively dissipating energy and driving the material’s self-repair. The study by Vakili et al. [96] provides a prime example of this. As shown in Figure 3d, they successfully constructed a physically cross-linked network based on host–guest inclusion interactions by grafting aminoazobenzene (AZO) onto gelatin chains (Gel-AZO) as the guest molecule and combining it with polycyclodextrin (PCD) as the host molecule. UV-Vis spectroscopy confirmed that, as the PCD concentration increased, the characteristic absorption peak of Gel-AZO at 360 nm shifted to a longer wavelength, indicating the successful formation of the host–guest complex. This dynamic network endowed the hydrogel with excellent self-healing capabilities: macroscopically, cut hydrogel fragments healed autonomously within 24 h of contact, whilst microscopic observations also revealed that interfacial cracks gradually disappeared over time. Rheological testing further revealed the dynamic properties of this system; the variation in the storage modulus (G′) and loss modulus (G″) with frequency indicates the disruption and reconstruction of the cross-linked network driven by host–guest interactions. Therefore, through the rational design of host–guest pairs, host–guest interactions can provide a robust, dynamic and reversible cross-linking platform for gelatin hydrogels, which is one of the key mechanisms for achieving their highly efficient self-healing. More importantly, through the careful design of host–guest pairs, stimulus-responsive properties can be further imparted to gelatin hydrogels. For example, the chemical state or conformation of certain guest molecules (such as ferrocene [97] and azobenzene) changes in response to external stimuli (e.g., redox potential or light), thereby altering their binding strength to the host molecules. This enables precise regulation of the hydrogel’s macroscopic properties and on-demand gel–sol transitions, greatly expanding its application potential in fields such as smart drug delivery and controllable tissue engineering scaffolds.

#### 3.3.2. Dynamic Covalent Bonds

Boronate bonds are dynamic [98], reversible covalent bonds formed by the condensation reaction between boronic acid and a diol. In the design of gelatin-based self-healing hydrogels, this bond serves as a core cross-linking unit due to its dynamic reversibility. It can be constructed through a complexation reaction between borates and components containing diol structures within the gelatin or composite system, thereby synergising with the native gelatin framework and other cross-linking networks to form a multi-cross-linked structure, endowing the hydrogel with excellent self-healing properties and functional adaptability. Extensive research has confirmed the practicality of boric acid and its derivatives (such as phenylboronic acid and its polymers) in the design of self-healing hydrogels. As shown in Figure 3e, Xie et al. [99] constructed an injectable self-healing hydrogel by combining GelMA, cationic guar gum (CG) and borax. Its self-healing property primarily stems from the dynamic boronate bonds formed between boric acid and the diol groups on the CG chains. Studies have shown that this cross-linked network enables the hydrogel to rapidly recover its mechanical strength following structural damage: oscillatory strain recovery experiments demonstrated that severed hydrogel fragments could self-heal within minutes of contact and, once healed, could withstand tensile stress without fracturing. This work provides a compelling example of the construction of self-healing boronate-bonded gelatin hydrogels. As depicted in Figure 3f, Li et al. [91] utilised the complexation of borax with the diol groups of PVA to introduce dynamic boronate bonds into GelMA hydrogels. These bonds, acting in concert with multiple hydrogen bonds, not only serve as sacrificial bonds to significantly enhance the hydrogel’s tensile elongation (≈160%) and strength (≈130 kPa), but also confer the ability to self-heal autonomously at room temperature, achieving a stress-induced self-healing efficiency of up to 86%. This study demonstrates that introducing boronate bonds into biopolymer networks such as GelMA can effectively address their inherent brittleness and inability to self-heal. Furthermore, Ge et al. [100] constructed a gelatin-based hydrogel with a dual dynamic cross-linking structure by forming boronate bonds between oxidised dextran (ODex) diols and borax, combined with Schiff base bonds generated between gelatin amine groups and ODex aldehyde groups, demonstrating excellent self-healing performance (with the modulus rapidly recovering to baseline values following network disruption at 400% strain).

The imine bond (Schiff base bond) is formed by the condensation reaction between an amine and an aldehyde group, and is one of the most commonly used bonds in dynamic covalent chemistry. This bond is highly regarded for its inherent self-healing properties in hydrogel systems; it facilitates frequent breakage and regeneration, thereby conferring immense application potential. The abundance of amino groups on gelatin chains makes it an ideal biopolymer for constructing imine-bonded self-healing hydrogels [101]. A major limitation of purely natural polymer-based self-healing hydrogels (SHHs) is the difficulty in precisely tuning their properties, frequently resulting in inadequate mechanical strength. Consequently, imine-bonded SHHs with controllable mechanical properties have been developed, and their mechanisms have been extensively investigated. For instance, Ye et al. [102] constructed a series of imine-based dynamic hydrogels by regulating the aldehyde content of oxidised HA (oHA) and the type of amined gelatin. The study demonstrated that by altering the chemical structure of the precursor polymer (e.g., increasing the oxidation time of oHA from 2 h to 8 h), the G′ of the hydrogel could be precisely controlled, with values varying over a wide range from 620 Pa to 4476 Pa. Furthermore, all hydrogels exhibited rapid self-healing; following high-strain rupture, their modulus recovered almost completely within 1 min, and macroscopically severed hydrogel fragments re-fused and withstood tensile stress within 1 h. Furthermore, the work by Qi et al. [103] further confirms the crucial role of imine bonds in the construction of multifunctional, high-strength self-healing hydrogels. By forming imine bonds between gelatin and protocatechuic acid (PA) whilst simultaneously introducing boronate bonds, they constructed a GPB hydrogel with dual dynamic cross-linking. This dual-network structure significantly enhances the material’s mechanical properties, with a maximum tensile strength of 21.37 kPa and a fracture toughness of 16.27 kJ/m^3^. When severed, the hydrogel can recover to 77% of its original tensile strength within one hour, demonstrating excellent self-healing efficiency, attributed to the breaking and reformation of dynamic covalent bonds, such as imine bonds, as illustrated in Figure 3g. Collectively, these findings demonstrate that imine bonds, as dynamic covalent bonds, are not only central to the rapid self-healing of hydrogels but also that their synergistic interactions with other components provide a strategy for precisely regulating mechanical properties. This enables the material to adapt perfectly to joint movement and self-repair in real time following injury, offering considerable potential for application in the management of sports injuries.

The Diels–Alder (DA) reaction is a reversible equilibrium involving the formation of a thermoreversible DA ring between a conjugated diene and a diene-acceptor reagent during a sol–gel process under heated conditions. Due to the high selectivity of the reactants in the DA reaction system and the fact that catalysts are typically involved in the reaction, no by-products are formed [88]. Consequently, the DA reaction is also referred to as a ‘click chemistry’ reaction. However, in traditional DA reactions used to construct gelatin-based self-healing hydrogels, the reverse reaction typically requires high temperatures exceeding 130 °C, which limits their biomedical applications. To resolve this issue, Wang et al. [104] introduced HPASi, a hyperbranched crosslinker containing multiple hydrogen bonds, to construct a secondary network on top of the primary covalent network formed via the DA reaction. The flexibility and hydrogen-bonding capabilities of HPASi significantly reduced the energy barrier for DA bond reconfiguration, lowering the activation temperature from 130 °C to 65 °C. Under these mild conditions, the hydrogel exhibited rapid shape recovery (3 s) and excellent cycling stability; simultaneously, MTT assays confirmed a 3T3 cell survival rate exceeding 100%, indicating good biocompatibility. This strategy, through synergistic effects, drives the reaction temperature closer to the physiological temperature of 37 °C, thereby better adapting to more demanding biomedical applications, such as skin wound repair and tissue-engineering scaffolds.

### 3.4. Gelatin-Based Smart Responsive Hydrogels

Smart, responsive hydrogels are a class of functional materials capable of sensing changes in the external microenvironment (physical, chemical, and biological signals) and dynamically adjusting their properties (such as swelling, contraction, shape deformation, modulation of mechanical properties, and the release of biomolecules). Their core advantage lies in their ability to mimic the dynamic characteristics of the natural ECM, thereby enabling precise adaptation to and active regulation of the microenvironment during tissue repair [105]. Due to their excellent biocompatibility, degradability, and cell-adhesion properties, gelatin and its derivatives are frequently used as matrix materials in combination with responsive components to construct smart hydrogels suitable for the repair of sports injuries.

#### 3.4.1. Gelatin-Based Physically Responsive Hydrogels

Thanks to their unique stimulus responsiveness and tunable physicochemical properties, physically responsive hydrogels are increasingly serving as a bridge between materials science and life sciences. These polymeric materials are capable of sensitively detecting and responding to changes in the external physical environment, such as temperature fluctuations, light intensity, electric and magnetic fields, and mechanical stress. By modulating their own structure, morphology or function, they achieve dynamic interaction with the external environment [106].

**Figure 4:**
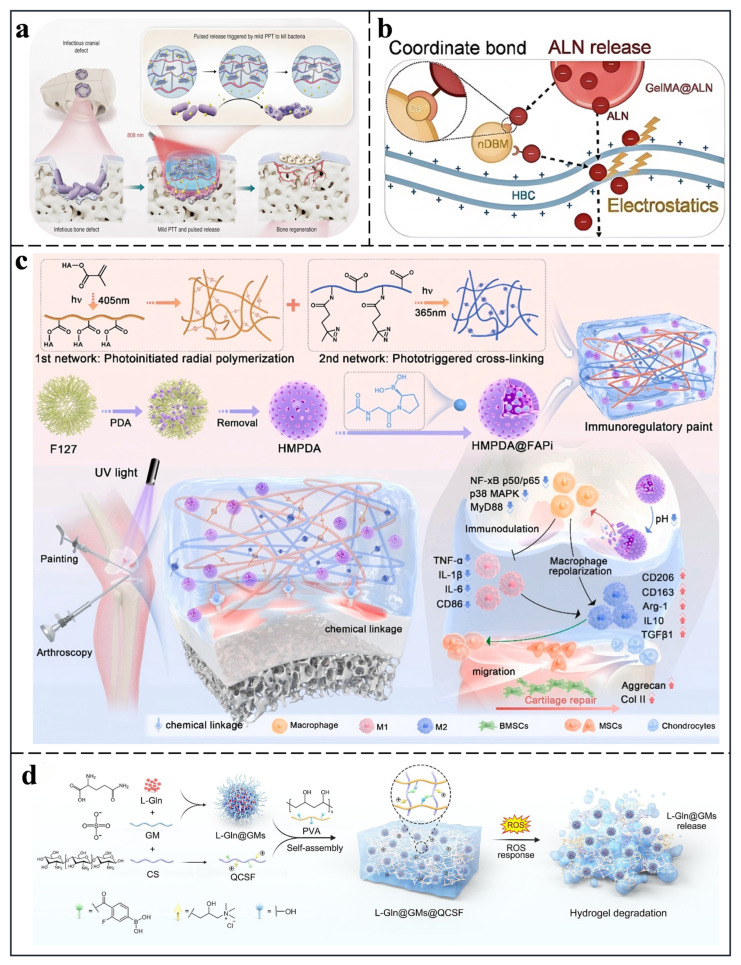
(**a**) Photothermal-sensitive nano composite hydrogel for repairing infectious bone defects in rats [82]. (**b**) Schematic illustration of the sustained-release mechanism of ALN in the HBC-nDBM-GelMA@ALN hydrogel [108]. (**c**) Schematic illustration of the preparation process for the H2G5@FDA composite hydrogel and its regulatory mechanism for arthroscopic cartilage repair [115]. (**d**) Preparation process of an injectable ROS-responsive self-assembling hydrogel for the treatment of osteoarthritis, which is loaded with L-glutamine [116].

Thermoresponsive hydrogels, also known as temperature-sensitive hydrogels, undergo a phase transition in response to changes in temperature. Depending on the phase transition process, they can exhibit either a low critical solution temperature (LCST) or a high critical solution temperature (UCST). The LCST is the temperature at which a thermoresponsive polymer undergoes a phase transition: above the LCST, the polymer undergoes a solution-to-gel transition, whilst below the LCST, the gel transforms into a liquid. This phenomenon is attributed to the interaction between hydrophilic chains and water molecules at low temperatures, leading to the dissolution of water [107]. However, as the temperature rises, hydrophobic interactions between hydrophobic groups become dominant, leading to aggregation and the formation of a cross-linked network, resulting in gelation. Many raw materials are used to prepare thermoresponsive hydrogels, and thermoresponsive composite hydrogels made from synthetic and natural polymers have been reported to perform well as drug carriers. Wu et al. [82] constructed a thermoresponsive hydrogel, GNA, by compositing GelMA with the thermoresponsive polymers N-isopropylacrylamide (NIPAM) and acrylamide (AAM). The thermoresponsive phase transition can be optimised by adjusting the GelMA concentration: at a GelMA concentration of 20%, the hydrogel exists in a porous sol state at room temperature, but undergoes significant shrinkage when the temperature rises to 45 °C (above the LCST), with porosity decreasing by 14.5% compared to room temperature. This phase transition stems from a temperature-dependent switch between hydrophilic and hydrophobic interactions and can be synergistically controlled with mild photothermal effects to achieve pulsed release of the loaded drug berberine (Figure 4a). In another similar study, Lei et al. [108] co-incorporated GelMA microspheres loaded with alendronate sodium (ALN) and nano-bone matrix (nDBM) into a hydroxybutyl chitosan (HBC) thermoresponsive hydrogel. This hydrogel exhibits typical LCST characteristics; at low temperatures, the HBC chains interact with water molecules via hydrogen bonding, maintaining the material in an injectable sol state; when the temperature rises to physiological body temperature (37 °C), the hydrogen bonds are broken, hydrophobic interactions become dominant, triggering cross-linking and entanglement of the molecular chains, thereby rapidly inducing a sol–gel phase transition at the injection site. This fixes active components, such as GelMA microspheres, at the site of the bone defect and, by leveraging this stable gel network, enables the controlled release of ALN and BMP-2 over several weeks (Figure 4b). Furthermore, thermosensitive hydrogels also hold promise as cell carriers. Although a large number of thermoresponsive gels are currently under development, only a few products have been commercialised to date, primarily due to a lack of long-term biocompatibility studies and patient compliance [107].

Photo-responsive hydrogels are stimuli-responsive materials that undergo rapid chemical or physical transformations upon exposure to specific light sources, commonly including ultraviolet (UV), visible (Vis), and near-infrared (NIR) light. Although UV cross-linking remains a common method, the cytotoxic effects of UV on cells and tissues have led researchers to favour Vis and NIR [109]. There are three primary mechanisms by which photoresponsive hydrogels exhibit characteristic changes in response to light stimulation: firstly, the chemical properties of photosensitive molecules change upon absorption of photons, triggering a phase transition that leads to alterations in the hydrogel’s volume, colour and other properties. Pourbadiei et al. [110] developed a gelatin-based interpenetrating network photoresponsive hydrogel that utilises ferric citrate as a physical cross-linking agent. This agent forms tridentate coordination complexes between Fe^3+^ and coordination sites on the gelatin chains, thereby imparting mechanical strength and stability to the hydrogel. Upon irradiation with visible light, Fe^3+^ undergoes a photoreduction reaction to Fe^2+^, causing the coordination bond strength to weaken and the coordination mode to shift from tridentate to monodentate or bidentate. This disrupts the crosslinked network, leading to increased gel swelling and a decline in mechanical properties. This strategy, based on light-controlled valence state conversion, enables non-contact, time-dependent control of the hydrogel’s degradation behaviour, providing a new approach for on-demand removal of smart wound dressings. Secondly, photosensitive substances absorb light energy and convert it into thermal energy, raising the gel temperature to the phase transition temperature and triggering changes in its physical properties. The Gel-TA-Fe^3+^ hydrogel prepared by Yang et al. [111] exhibits near-infrared (NIR) thermoresponsive properties due to the incorporation of Fe^3+^ as a cross-linking agent. Upon irradiation with 808 nm NIR light, the coordination structures formed between Fe^3+^ and phenolic hydroxyl groups within the hydrogel efficiently absorb light energy and convert it into thermal energy, thereby raising the gel system’s temperature. This temperature rise is controllable and remains within the range tolerable to the human body, thereby enabling photothermal antimicrobial therapy and promoting the healing of infected wounds. The third mechanism involves the decomposition of photosensitive molecules in the hydrogel under light irradiation, generating a large number of ions. This leads to a difference in ion concentration between the interior and exterior of the hydrogel, causing a sudden change in osmotic pressure that triggers a reaction or a change in the hydrogel network, ultimately leading to the hydrogel expanding or contracting [112]. He et al. [113] prepared a photosensitive GelMA hydrogel loaded with AgNPs using a blue-light-mediated LAP-induced technique. This material demonstrated excellent broad-spectrum antibacterial activity and angiogenic properties, demonstrating the application value of responsive gelatin-based hydrogels in the construction of sports injury scaffolds that combine anti-infection properties with the promotion of tissue reconstruction.

#### 3.4.2. Gelatin-Based Chemically Responsive Hydrogels

Chemically responsive hydrogels are characterised by the incorporation of specific functional groups and cross-linking structures into their architecture. Such hydrogels typically contain acidic, basic, ionic or reductive groups, which undergo reversible or irreversible chemical changes in response to external chemical stimuli, thereby modulating their physical properties (such as swelling capacity and permeability) or chemical state (such as charge and reactivity). For example, by incorporating pH-sensitive carboxyl, amino or sulphonic acid groups, hydrogels can respond to local pH changes, enabling controlled drug release or the regulation of cellular behaviour. Through careful chemical design and modification, chemically responsive hydrogels not only enhance the functionality of the hydrogel but also provide more flexible and precise material options for biomedical applications. pH-responsive and ROS-responsive hydrogels are currently the two most extensively studied types of chemically responsive hydrogels [114].

pH-responsive hydrogels are among the most extensively studied types of smart, responsive hydrogels. These hydrogels typically contain pH-sensitive acidic and basic groups, including carboxyl and amino groups, or pH-sensitive dynamic covalent bonds, which enable the controlled release of drugs. Under pathological conditions, changes in the pH of human tissues cause the dissociation of ionisable acidic and basic groups, thereby disrupting the hydrogen bonds and electrostatic interactions between macromolecular chains within the hydrogel network. This ultimately leads to the contraction or expansion of the hydrogel, thereby controlling drug release. A second mechanism leading to hydrogel degradation is the dissociation of dynamic covalent bonds within the hydrogel [106]. As shown in Figure 4c, Jia et al. [115] reported a gelatin-based smart hydrogel cross-linked via carbene chemistry and covalently incorporating drug-loaded hollow mesoporous polydopamine nanoparticles (HMPDA@FAPI). In the acidic inflammatory microenvironment of cartilage injury (pH~5.5), the HMPDA nanostructure undergoes responsive degradation, thereby precisely controlling the release of the anti-inflammatory drug FAPI to modulate the local immune response and promote cartilage regeneration. This work demonstrates a strategy for constructing smart gelatin hydrogels by introducing pH-responsive nanostructures to enable site-specific, on-demand drug delivery at the lesion site.

ROS-responsive hydrogels are capable of sensing and responding to changes in the levels of ROS in their environment, as evidenced by alterations in their solubility or the degradation of chemical bonds. ROS are chemically derived oxygen molecules, including free radicals such as superoxide, hydroxyl and peroxyl radicals, as well as non-radicals such as hypochlorite, ozone, singlet oxygen and hydrogen peroxide. Moderate levels of ROS are essential for various biological functions, such as cell signalling, hormone production and the regulation of protein function. However, excessive ROS is associated with a range of diseases. Consequently, ROS-responsive hydrogels are well-suited for the development of stimulus-responsive biomaterials for targeted therapies [106]. As illustrated in Figure 4d, research by Wang et al. [116] demonstrates that ROS-responsive hydrogels constructed using dynamic phenylboronate bonds can effectively load drugs (such as L-glutamine encapsulated in gelatin microspheres) and undergo bond cleavage upon stimulation by ROS such as hydrogen peroxide (H_2_O_2_), leading to gel degradation and controlled drug release behaviour. This provides a materials science foundation for achieving targeted therapy in inflammatory diseases such as osteoarthritis. In further research, Guo et al. [117] combined ROS-responsiveness with enzyme-responsiveness to report a HA hydrogel based on dynamic boronate bonds. By embedding drug-loaded gelatin microspheres, the study established a dual-responsive mechanism: the boronate bonds within the gel network degrade in response to ROS, whilst the gelatin carriers are specifically degraded by MMP-9/MMP-13, which are highly expressed in pathological environments, thereby synergistically achieving targeted controlled drug release. This design enhances the precision of drug release and endows the material with physical properties such as lubricity, demonstrating the development potential of smart hydrogels in functional integration and synergistic therapy.

In existing research, the types of responses exhibited by smart hydrogels have been further expanded to include glucose-responsive and CO_2_-responsive properties. Glucose-responsive hydrogels are often based on mechanisms such as glucose oxidase catalysis, lectin competitive binding, or reversible interactions between phenylboronic acid and diols; they can sense changes in local glucose concentrations and are suitable for regulating the metabolic microenvironment during diabetes-related tissue repair. In recent years, the concept of CO_2_-responsive hydrogels has also been introduced. These refer to a class of hydrogels that undergo structural or performance transformations by altering the solution pH via the addition or removal of CO_2_ gas. They possess green stimulus properties and are non-toxic and harmless, providing a new material strategy for targeted delivery and regulation of the tissue microenvironment under conditions of respiratory or metabolic abnormalities [106].

### 3.5. Gelatin-Based Adhesive Hydrogels

As the applications of hydrogels in the biomedical field continue to expand, tissue-adhesive hydrogels have become a research focus in sports injury rehabilitation due to their ability to closely conform to biological tissues, provide mechanical support, and promote repair. An ideal adhesive hydrogel must not only possess excellent biocompatibility and mechanical compatibility, but also be capable of achieving stable, controllable adhesion in a moist, dynamic physiological environment to meet the requirements of different stages of rehabilitation following sports injuries [118]. Gelatin, a naturally derived protein, is an ideal candidate for the construction of smart adhesive hydrogels due to its excellent biocompatibility, degradability, and abundance of reactive functional groups. The following subsections summarise the adhesion mechanisms and design strategies for gelatin-based bioadhesive hydrogels [119].

#### 3.5.1. Chemical Bonds

**Figure 5:**
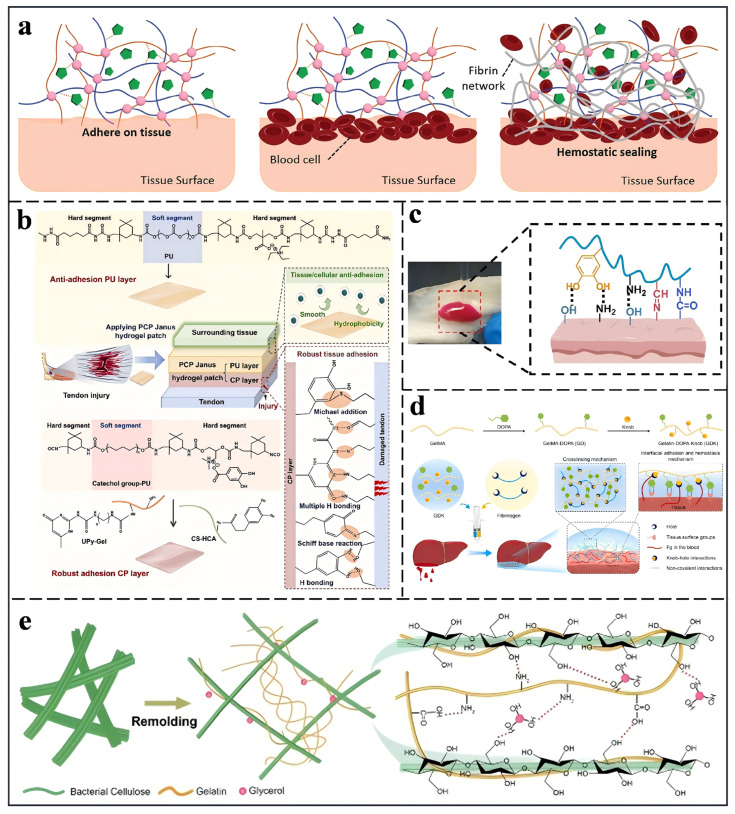
(**a**) Schematic illustration of AOT adhesion to tissue surfaces and its application to bleeding wounds. Orange: AGel; Blue: OHA; Pink: H-bond sites; Green: TA; Red: RBCs; Gray: fibrin network; Light pink: tissue [121]. (**b**) Schematic illustration of the preparation of Janus hydrogel patches and the mechanism of tendon repair [122]. (**c**) Schematic illustration of the adhesion mechanism between GMODV hydrogel and tissue [123]. (**d**) The preparation process, cross-linking mechanism, and mechanisms of tissue adhesion and haemostasis of the GDK/Fg hydrogel [126]. (**e**) Schematic diagram illustrating the hydrogen bond reorganisation and topological network formation mechanisms of the bacterial cellulose–gelatin-based composite hydrogel [129].

Chemical bonds formed via reactive ester groups or Schiff base reactions are among the primary adhesion mechanisms in the design of adhesive gelatins. These covalent bonds form through chemical interactions between specific reactive groups on the hydrogel surface and complementary groups on the tissue surface [120]. For example, in Schiff base bonds, reversible bonds form between an amine and an aldehyde, thereby conferring reversibility. The abundance of amine and aldehyde groups in human tissue, coupled with the dynamic nature of Schiff base bonds, enhances the value of this reaction for tissue adhesion. The research by Wang et al. [121] provides a typical example of the design of gelatin-based adhesive hydrogels based on the Schiff base reaction. The core mechanism of adhesion of this hydrogel relies on the rapid formation of dynamic Schiff base bonds between the amino groups of aminated gelatin and the aldehyde groups of aldehyde-modified hyaluronic acid, whilst simultaneously constructing a multi-hydrogen bond network under the synergy of tannic acid, thereby collectively endowing the hydrogel with excellent wet tissue adhesion (adhesion strength reaching 48.67 ± 0.16 kPa) and rapid gelation capability (Figure 5a). This study confirms that introducing Schiff base reactions into the gelatin network through appropriate chemical modification is an effective strategy for constructing functional hydrogels capable of achieving immediate, robust adhesion in moist physiological environments. It also suggests that such adhesive hydrogels have potential for managing acute vascular or tissue injuries associated with sports-related trauma.

Mussel chemistry based on catechol functionality represents another emerging method of chemical bonding; it exhibits strong adhesion in humid environments, providing significant inspiration for the development of high-performance adhesive hydrogels suitable for moist tissue environments. The dopamine and catechol structures abundant in mussel byssus protein can form covalent and non-covalent synergistic interactions with various functional groups on tissue surfaces via their hydroquinone groups, thereby significantly enhancing the material’s adhesion strength and stability under wet conditions [119]. In recent years, researchers have introduced dopamine into gelatin-based hydrogel networks, not only enhancing their adhesion to soft tissues (such as muscles and tendons, Figure 5b) but also promoting the adhesion and migration of fibroblasts and endothelial cells through the interaction of catechol groups with extracellular matrix proteins, thereby accelerating the tissue repair process [122]. For example, a gelatin–ODex dual-network hydrogel constructed via a synergistic combination of Schiff base reaction and photocrosslinking exhibited approximately a twofold increase in adhesion strength following the introduction of dopamine (Figure 5c). It demonstrated good biocompatibility and low haemolytic activity in both in vitro and in vivo experiments, suggesting potential applications in the repair of superficial or deep tissues resulting from sports injuries. Such hydrogels not only enable rapid wound closure and haemostasis but also further promote angiogenesis and tissue regeneration through the sustained release of growth factors (such as VEGF), thereby demonstrating significant potential for application in sports medicine and rehabilitation engineering [123].

#### 3.5.2. Physical Binding

Physical binding, driven by weak intermolecular interactions, can provide sufficient adhesion without causing discomfort to the tissue. Such interactions include van der Waals forces, π-π stacking, ionic bonds, hydrogen bonds, and hydrophobic interactions; their cumulative effects can synergistically achieve strong, durable adhesion and typically do not require complex chemical reaction steps, thereby simplifying preparation [124]. Among these, the catechol group is one of the key functional units for achieving physical binding; its resorcinol structure can bind to tissue surfaces through various non-covalent interactions, such as hydrogen bonding, π-π interactions and metal coordination. However, the effectiveness of these interactions depends heavily on the precise regulation of the catechol group’s oxidation state [125]. To further enhance the strength and specificity of physical adhesion, researchers often combine the action of catechol groups with biomimetic molecular recognition mechanisms. As shown in Figure 5d, Liu et al. [126] designed a gelatin-based physically cross-linked adhesive hydrogel, the core adhesion mechanism of which relies on the synergy of two physical interactions: firstly, biomimetic ‘protrusion-pore’ specific recognition, achieved by grafting fibrinogen ‘protrusion’ peptides onto gelatin, enabling rapid, high-affinity specific binding with the ‘pore’ structures on fibrinogen; secondly, catechol-mediated non-covalent interactions, wherein the introduced dopamine, via its catechol groups, enhances cohesion through hydrogen bonding and π-π stacking, whilst extensively interacting with various functional groups on the surface of moist tissues, thereby achieving strong interfacial adhesion. This synergistic strategy significantly enhances the hydrogel’s adhesive performance, with the lap shear strength increasing to 43.9 ± 3.9 kPa—approximately 44 times that of commercial fibrin glue. This demonstrates that integrating biomimetic recognition with catechol-mediated physical interactions enables the construction of a robust, rapid, and non-covalent physical adhesion network within gelatin-based hydrogels.

Another important non-covalent bond in adhesive hydrogels is the host–guest interaction. This strategy utilises the cavities of macrocyclic host molecules (such as cyclodextrins) to reversibly encapsulate specific guest molecules, forming a dynamic, physical cross-linking network within the material and at the interface between the material and tissue [119]. For instance, Zeng et al. [127] developed a multifunctional supramolecular hydrogel for complex wound repair, in which the core physical cross-linking of the three-dimensional network stems from host–guest interactions. The researchers achieved this by acryloylating β-cyclodextrin to facilitate host–guest recognition and inclusion with aromatic amino acid residues on the gelatin chains, followed by mild photo-cross-linking to construct a stable gel network. This dynamic behaviour, driven by physical adhesion mechanisms, enables the hydrogel to adapt to irregular wound surfaces whilst maintaining long-term structural integrity. It protects the wound and guides orderly tissue regeneration in dynamic stress environments, thereby enhancing the quality of recovery.

#### 3.5.3. Topological Bonding

Topological bonding is another highly effective mechanism that confers excellent adhesion properties on hydrogels. This mechanism does not rely on chemical reactions involving specific functional groups, but rather utilises the diffusion, penetration and topological entanglement of flexible polymer chains at the interface to achieve a ‘molecular stitching’ effect, thereby forming a robust bond on moist, dynamic tissue surfaces [128]. For example, Zhou et al. [129] designed a gelatin-based composite hydrogel with excellent comprehensive properties using a hydrogen-bond topological remodelling strategy. The enhancement of its adhesion and cohesion strengths is largely attributable to the increased topological entanglement. As illustrated in Figure 5e, to achieve topological remodelling, the researchers introduced gelatin and glycerol into the bacterial cellulose network to act as hydrogen-bond modulators. These substances partially dissociated the dense hydrogen bonds between cellulose chains, causing them to untangle and release ultrafine nanofibres. These newly formed ultrafine fibres reconnected with gelatin chains and glycerol molecules via numerous hydrogen bonds, forming a physical network characterised by high interpenetration and microscopic-scale topological entanglement. This microstructure, dominated by a topological bonding mechanism, not only significantly enhances the gel’s intrinsic toughness and tensile strength but also confers it with remarkable tissue adhesion properties [130]. Biologically, such robust tissue integration provides an indispensable physical template for ECM remodeling and host angiogenesis. Because the gelatin matrix retains cell-responsive Arg-Gly-Asp (RGD) sequences, the adhered hydrogel specifically binds to integrin receptors on migrating host cells, creating a biomimetic temporary scaffold. This supports synchronized matrix degradation and host cell infiltration. Concurrently, this stabilized microenvironment recruits vascular endothelial cells, significantly promoting endothelial sprouting and functional angiogenesis. Ultimately, this cascade orchestrates a seamless transition from the artificial hydrogel to mature, vascularized native musculoskeletal tissues [131]. Furthermore, the hydrogel exhibits excellent transparency, long-lasting moisture retention and outstanding biocompatibility. In the context of sports injury rehabilitation, such gelatin-based adhesives reinforced by physical topological entanglement—which require no introduction of exogenous chemical cross-linking agents or ‘sutures’ that may trigger immune responses or cytotoxicity—can adhere closely, durably and safely to irregular or mobile wound surfaces. This provides a solution for tissue protection during dynamic rehabilitation that combines both high strength and superior biosafety [128,132].

In summary, Section 3 systematically delineates how the strategic integration of functional additives and tailored crosslinking networks equips gelatin hydrogels with distinct bio-features, specifically electrical conductivity, antimicrobial action, autonomous self-healing, tissue adhesion, and smart responsiveness. To establish a clear blueprint before exploring their tissue-specific therapeutic roles, Table 2 comprehensively consolidates the chemical formulations, target biological performance, and inherent engineering trade-offs of these multi-functional platforms.

## 4. Applications of Gelatin-Based Multifunctional Hydrogels in the Repair of Sports Injuries

### 4.1. Repair of Bone and Articular Cartilage Injuries

Sports-related bone and articular cartilage injuries are particularly common in competitive sports and high-intensity training. Bone injuries include traumatic fractures, stress fractures and bone contusions; articular cartilage injuries manifest in various forms, ranging from superficial wear to full-thickness defects, and often extend into the subchondral bone, resulting in osteochondral defects that are difficult to repair. Such injuries not only cause pain and functional impairment, but inadequate repair may also lead to the progression of osteoarthritis, severely affecting athletic performance and quality of life [133]. Bone healing involves a complex process of inflammation, proliferation and remodelling, and is prone to delayed healing or non-union due to insufficient blood supply or mechanical instability. Articular cartilage, lacking vascular, neural and lymphatic supply, has extremely limited self-repair capacity. Current clinical approaches, such as surgical fixation and microfracture techniques, whilst alleviating structural issues, struggle to achieve true tissue regeneration; pharmacological treatments offer only symptomatic relief; and transplantation faces challenges including limited donor sources, immune rejection and functional mismatch [134]. Consequently, the development of novel repair materials that combine biocompatibility, degradability, mechanical adaptability and biological activity has become a key focus for promoting the efficient repair of sports-related osteochondral injuries.

**Figure 6:**
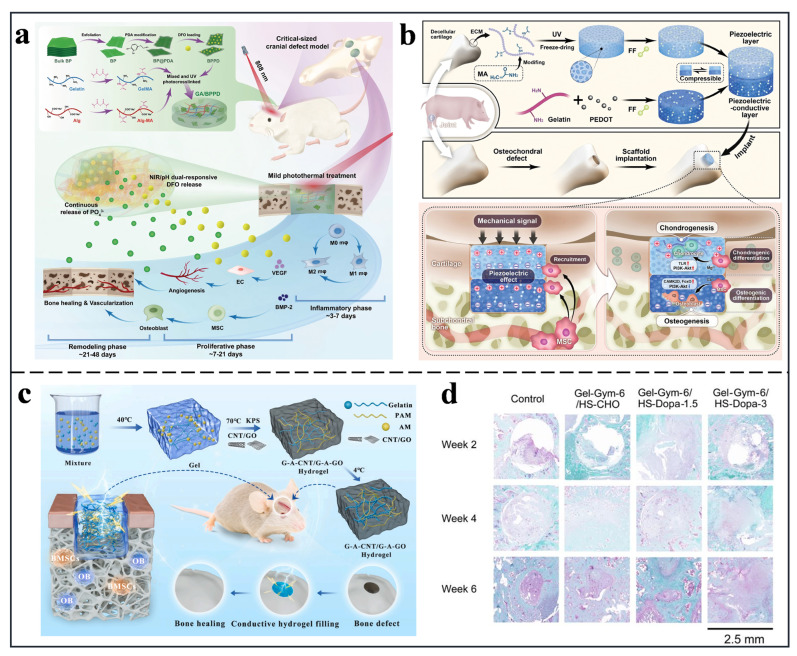
(**a**) Smart NIR/pH dual-responsive therapeutic system for accelerated bone regeneration [135]. (**b**) Biodegradable piezoelectric–conductive scaffold for osteochondral defect reconstruction [136]. (**c**) G-A-CNTs/G-A-GO conductive hydrogel filling for cranial bone healing under electrical stimulation [137]. (**d**) Histological staining results of hydrogel groups at weeks 2, 4, and 6 [138].

Gelatin-based multifunctional hydrogels offer a multidimensional repair strategy for such injuries by integrating properties such as smart responsiveness, enhanced conductivity and adhesive self-healing. For example, in response to the ROS and acidic microenvironment following fractures or cartilage defects, ROS/pH-responsive gelatin hydrogels can be designed to enable the targeted release of anti-inflammatory or osteogenic drugs, thereby precisely regulating local immune responses. As shown in Figure 6a, targeting the ROS/pH microenvironment, Wu et al. [135] prepared black phosphorus nanosheets (BPPD) loaded with difenoxin (DFO) and incorporated them into a GelMA/sodium alginate (GA) hybrid hydrogel. BPPD serves as both a bioactive component and a near-infrared (NIR) photothermal agent, endowing the hydrogel with excellent NIR/pH dual-responsive properties and enabling the stimulus-responsive release of DFO and PO_4_^3−^ during bone regeneration. Under the action of mild NIR-triggered photothermal therapy, the hydrogel demonstrated positive effects in promoting osteogenesis and angiogenesis, scavenging excessive ROS, and inducing macrophage polarisation to the M2 phenotype; furthermore, through the M2-polarised bone immune microenvironment, it further drove the secretion of angiogenesis- and osteogenesis-related cytokines, thereby significantly accelerating bone healing in a rat cranial marginal defect model. Meanwhile, conductive gelatin hydrogels doped with conductive components (such as PEDOT, GO or CNTs) can mimic the physiological electrical microenvironment of bone tissue, significantly promoting the osteogenic differentiation and mineralisation of BMSCs when subjected to exogenous electrical stimulation. Liu et al. [136] developed a biodegradable piezoelectric–conductive integrated hydrogel scaffold for the repair of osteochondral defects. As shown in Figure 6b, the scaffold features a dual-layer design: the upper layer consists of a piezoelectric layer derived from decellularised cartilage extracellular matrix modified with diphenylalanine, whilst the lower layer comprises a PEDOT-reinforced conductive gelatin layer. During joint movement, mechanical pressure causes the upper layer to deform and generate a positive charge, attracting BMSCs to migrate and promoting their chondrogenic differentiation; the lower layer accumulates a negative charge, inducing stem cells to differentiate in an osteogenic direction. This hydrogel generates electrical stimulation during movement without an external power source and demonstrates excellent repair effects in a bone-cartilage defect model in Baima pigs. The figure illustrates the mechanism of action of this scaffold, clearly demonstrating how the bilayer structure generates an electric potential difference under pressure and guides bidirectional cell differentiation. It exemplifies the intelligent design of the gelatin hydrogel as a carrier, combining conductive and piezoelectric properties to achieve bone and cartilage repair. In another study, Geng et al. [137] developed a conductive composite hydrogel based on a gelatin–polyacrylamide double network doped with CNTs for the promotion of bone defect repair via microcurrent stimulation (Figure 6c). Simulation and experimental results confirmed that CNTs form a more efficient conductive network than GO, significantly enhancing the hydrogel’s electrical conductivity (reaching 4.2 × 10^−5^ S/m at 0.75 wt%) and mechanical properties (with a tensile strength of 0.23 MPa). This hydrogel exhibits good biocompatibility; when implanted into rat cranial defects, it significantly promotes the osteogenic differentiation and mineralisation of BMSCs under applied microcurrent stimulation, ultimately achieving complete defect healing (with the 0.75% CNT-doped group showing near-complete defect coverage at 12 weeks).

Furthermore, self-healing and tissue-adhesive hydrogels based on dynamic covalent bonds can be injected via minimally invasive procedures to conform to irregular bone and cartilage defects, maintaining structural integrity during joint movement and providing stable three-dimensional scaffolding for cell infiltration. Yang et al. [138] developed a dopamine-functionalised gelatin/chondroitin sulphate composite hydrogel that exhibits excellent self-healing and tissue-adhesive properties, is injectable, and gels rapidly under UV light. This hydrogel not only exhibits good biocompatibility and anti-inflammatory properties in vitro, but also significantly promotes skin wound healing and bone defect repair in rat models, restoring the animals’ motor function (Figure 6d). Particularly for weight-bearing joints subjected to high-impact sports, such as the knee or ankle, early intervention using these biomaterials is critical. Recently, researchers have developed specialized load-bearing gelatin-based scaffolds specifically to treat focal osteochondral defects—a typical injury among professional basketball and soccer players caused by repetitive jumping and pivoting. By precisely matching the compressive modulus of native cartilage, these biomimetic hydrogels effectively absorb massive mechanical shocks, thereby preventing the progressive post-traumatic osteoarthritis frequently observed in athletic populations [139].

### 4.2. Repair of Tendon and Ligament Injuries

As key transmission tissues connecting muscles to bones, tendons and ligaments are subjected to high-intensity tensile loads over extended periods. In competitive sports, the inherent contradiction between their ‘low metabolic rate’ and ‘high mechanical demands’ makes them highly susceptible to the formation of disorganised fibrous scar tissue with poor mechanical properties following injury. This results in athletes facing an extremely high risk of re-rupture during high-intensity training [140]. Critically, most current hydrogel models fail to replicate the complex, cyclic triaxial loading experienced by native tendons. The severe shear stress at the tendon–hydrogel interface often leads to premature scaffold failure before sufficient ECM deposition occurs. To address this challenge, researchers have integrated the strategies described in Section 3—self-healing fatigue resistance, strong interfacial adhesion, and smart, responsive release—to resolve three major clinical challenges: ‘dynamic mechanical adaptation’, ‘reconstruction of the tendon-bone interface gradient’, and ‘restoration of microenvironmental balance’.

**Figure 7:**
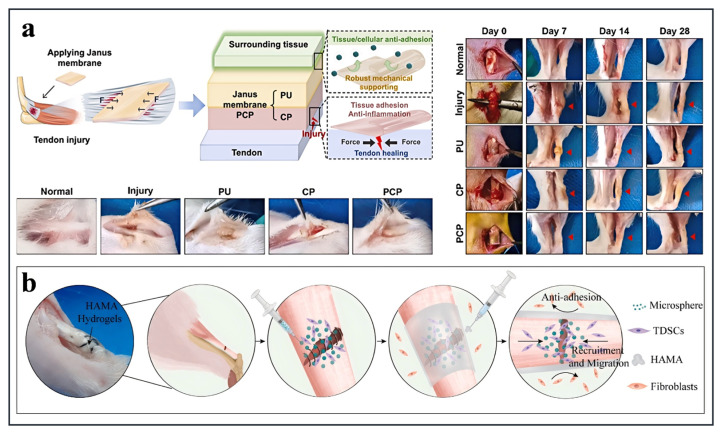
(**a**) Schematic diagram of the rat tendon injury model and photographs showing post-operative adhesion in the tendon wound area for each group [122]. (**b**) Mechanism of action of the composite microsphere-hydrogel membrane in promoting endogenous tendon healing and preventing exogenous adhesion [142].

Firstly, regarding the issues of material fatigue and fracture caused by repetitive cyclic stress during rehabilitation training, the energy-dissipation mechanism based on ‘non-covalent interactions’ discussed earlier has been shown to be key to enhancing the scaffold’s toughness. Tan et al. [122] ingeniously utilised the multiple hydrogen bonds formed between UPy groups to construct a dynamically adaptive Janus hydrogel patch. During dynamic stretching, these dynamic hydrogen bonds act as ‘sacrificial units’, breaking preferentially to dissipate mechanical energy and inducing strain-induced crystallisation in the polymer chains, thereby endowing the material with exceptional fatigue resistance and self-healing capabilities. This molecular design not only addresses the inherent brittleness of conventional hydrogels but also activates cellular regeneration pathways through the transmission of mechanical signals (Figure 7a). Secondly, during high-demand surgical interventions such as ACL reconstruction or rotator cuff tear repair—which are paradigmatic sports injuries requiring massive load-bearing capacity—simple ‘chemical adhesion’ is often insufficient to resolve the profound modulus mismatch at the tendon–bone interface; the construction of biomimetic gradient structures has therefore emerged as an advanced strategy. Liu et al. [141] combined the biological activity of gelatin with ion-modulation strategies to construct a hydrogel scaffold containing a continuous gradient of bioactive glass ions using gelatin and modified sodium alginate. This design goes beyond traditional interfacial adhesion; by spatially modulating the release concentrations of Ca and Si ions, it precisely induces synchronous ‘osteogenic–chondrogenic’ differentiation at the interface. This biomimetic gradient structure successfully regenerated multi-layered transitional tissue, including a mineralised fibrocartilage layer, whilst significantly eliminating stress concentration at the interface. Finally, the key to functional recovery lies in accelerating tissue regeneration whilst preventing post-operative adhesions. In response to specific clinical needs, such as irregular defects and minimally invasive treatments, injectable repair systems offer broad application prospects due to their excellent tissue compatibility and minimally invasive nature. Yang et al. [142] developed a growth factor-capturing microsphere/hydrogel composite system. They designed GelMA microspheres loaded with platelet-derived growth factor and fibronectin, and encapsulated them within an anti-adhesion HAMA hydrogel. This system not only physically blocks fibroblast invasion via the outer HAMA layer but also actively recruits endogenous tendon stem cells via sustained PDGF-BB release from the microspheres, thereby promoting their migration and differentiation. This ‘internal recruitment and external defence’ strategy successfully balances the healing mechanisms, achieving high-quality, scar-free healing in an Achilles tendon rupture model (Figure 7b).

### 4.3. Muscle Injury Repair

Skeletal muscle (SM) accounts for over 40% of body weight and plays a crucial role in movement and respiration [143]. Although SM possesses an innate capacity to regenerate following minor contusions or mild exercise-induced micro-tears, severe, high-velocity sports injuries—such as grade III muscle strains (e.g., hamstring or quadriceps tears) and devastating impact traumas—frequently overwhelm this endogenous repair mechanism. This leads to massive structural deficits and irreversible loss of muscle function, clinically defined as volumetric muscle loss (VML). Currently, autologous muscle tissue transplantation is the gold standard for the clinical treatment of VML; however, due to limitations in donor resources and regenerative efficiency, achieving optimal functional restoration remains challenging [6]. With recent advances in tissue engineering, researchers have developed various hydrogel materials for SM regeneration, and multifunctional hydrogels centred on gelatin have demonstrated the potential to overcome the limitations of conventional treatments.

**Figure 8:**
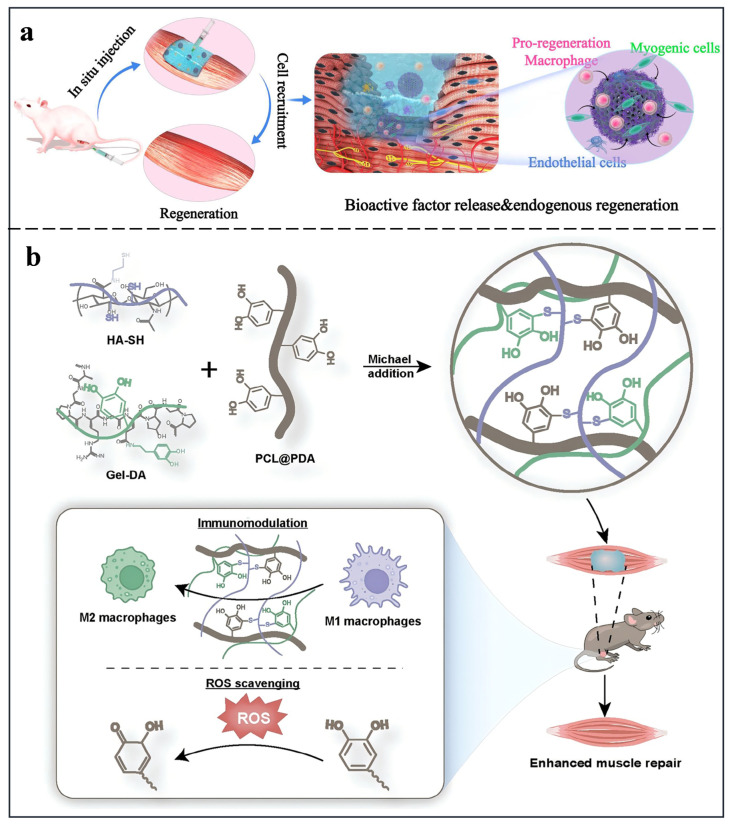
(**a**) In situ injection of the mECM@IL4+PM@IGF1 composite promotes endogenous muscle regeneration [144]. (**b**) Schematic diagram of the interfacially cross-linked hydrogel–poly(ε-caprolactone) nanofibre composite and its effect on the regenerative microenvironment during the repair of volumetric muscle defects [146].

Current research indicates that a well-designed three-dimensional hydrogel microenvironment can significantly improve cell adhesion and the quality of myofibre regeneration, thereby providing a foundation for optimising tissue repair. For example, Li et al. [144] developed an injectable composite system comprising elastic, porous PLCL microspheres and a myogenic extracellular matrix (mECM) hydrogel. This system, through its highly interconnected pore structure and ECM components, provides a favourable three-dimensional scaffold for cell infiltration and adhesion. In a severe 40% volumetric muscle loss model (defect size: 10 × 6 × 4 mm^3^), it significantly promoted the formation of new myofibres, reduced collagen deposition and enhanced muscle tissue reconstruction over an 8-week evaluation period, thereby validating the critical role of three-dimensional hydrogel microenvironment design in muscle injury repair (Figure 8a). Beyond severe VML, acute muscle tears and deep contusions resulting from intense eccentric contractions—such as hamstring strains during sprinting—represent another prevalent challenge in sports medicine. To address the rapid mobilization needs of athletes, recent studies have engineered injectable, highly stretchable gelatin hydrogels that perfectly conform to the dynamic contraction and relaxation of SM. These stretchable matrices not only mechanically bridge the torn myofibers but also significantly reduce the formation of fibrotic scar tissue, which is the primary culprit for recurrent muscle strains in track and field athletes [145]. It is worth noting that muscle injury sites are often accompanied by persistent inflammatory responses and abnormal accumulation of ROS, which inhibit myosatellite cell function and hinder effective tissue regeneration. To address this bottleneck, Yu et al. [146] developed a composite system formed by the covalent interfacial cross-linking of PDA-modified polycaprolactone nanofibres with a gelatin/hyaluronic acid hydrogel. As shown in Figure 8b, by regulating the material–cell interface, this system significantly alleviates local oxidative stress and promotes pro-regenerative macrophage polarisation. In the VML model, this hydrogel effectively reduced ROS levels and the inflammatory burden in the injured area, minimised fibrosis formation, and significantly improved the maturity of newly formed muscle fibres and functional recovery. This validated the feasibility of a strategy that utilises the material’s intrinsic interfacial chemical properties to modulate inflammatory responses and oxidative stress, thereby improving the muscle regenerative microenvironment. Building on this foundation, the functional applications of gelatin have been further extended into electrophysiology. Lin et al. [147] developed a conductive gelatin hydrogel that, by establishing electrical coupling with damaged muscle tissue and leveraging its inherent immunomodulatory properties to improve the chronic inflammatory microenvironment, effectively supported the regeneration and functional recovery of muscle fibers under diabetic conditions. Building on this, Jin et al. [148] proposed incorporating self-healing and adhesive properties into the conductive gelatin hydrogel, enabling it to withstand high-frequency deformation in moving areas and ensuring that the dressing remains firmly in place over the wound throughout the healing process, thereby significantly improving healing efficiency and safety.

### 4.4. Repair of Spinal Cord Injury

Spinal cord injury (SCI) is most commonly caused by trauma; the resulting neurological damage can severely impair motor, sensory and autonomic functions [149,150]. It is reported that there are at least 250,000 new cases of SCI worldwide each year. Although survival rates among SCI patients have risen steadily in recent decades, the associated health burden and economic costs continue to increase, and SCI remains a major clinical challenge [151]. Effective treatment of spinal cord injury requires controlling inflammation, promoting NSC recruitment, facilitating neuronal regeneration, and guiding the development of myelinated axons [152]. It is worth noting that the spinal cord is a soft, water-like biological structure with a stiffness range of 3–300 kPa. Hydrogels, as biomaterials, offer unique advantages in SCI repair due to their high hydrophilicity and compatible physical properties [153].

**Figure 9:**
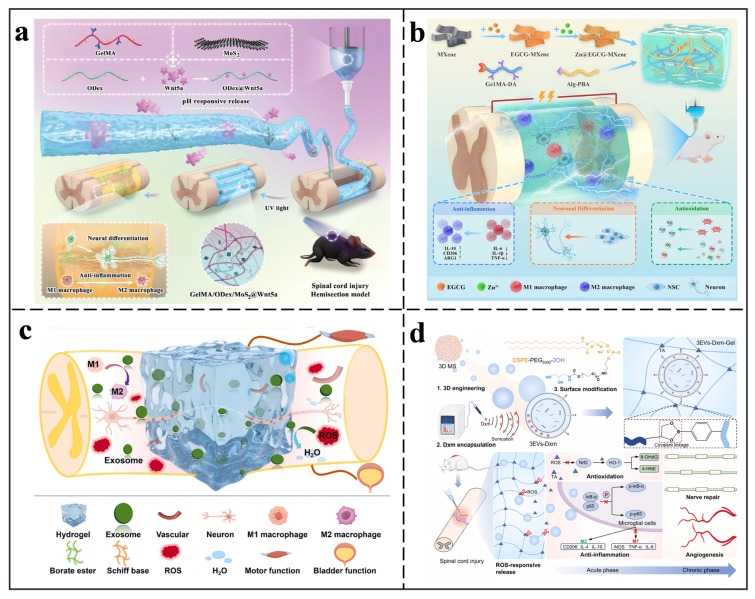
(**a**) Microenvironment-responsive injectable hydrogel promoting neural differentiation and anti-inflammatory effects [154]. (**b**) Conductive hydrogel combined with electrical stimulation for microenvironment remodelling [155]. (**c**) GEL-EXO composite scavenging ROS to promote functional recovery after SCI [156]. (**d**) Preparation process and therapeutic mechanism of the 3EVs-Dxm-Gel [151].

Currently, injectable polymer systems with excellent responsiveness are a hot topic in SCI repair research. Their key advantage lies in their ability to reversibly switch between sol and gel states in response to external stimuli, thereby enabling flexible filling of irregularly shaped cavities—a method that is more effective than direct hydrogel implantation. Furthermore, these systems can serve as carriers for therapeutic agents, such as drugs and growth factors, thereby accelerating the repair of spinal cord injuries [153]. As shown in Figure 9a, inspired by the pathological characteristics of the acidic microenvironment following spinal cord injury, Liu et al. [154] developed a microenvironment-responsive injectable gelatin-based hydrogel. This hydrogel utilises dynamic Schiff base chemistry to achieve rapid and reversible sol–gel transitions, enabling flexible filling of irregular defect cavities resulting from spinal cord injury. In a mouse model of 2 mm hemisection spinal cord injury, this hydrogel effectively reduced the cystic cavity in the injury zone and promoted the integration of injured tissue with the intact spinal cord. By utilising the acidic microenvironment at the site of acute spinal cord injury, it achieved on-demand release of Wnt5a via acid-sensitive cleavage of the Schiff base bond, rapidly suppressing the expression of pro-inflammatory factors and polarising macrophages towards the M2 phenotype, whilst simultaneously utilising the RGD motif of GelMA to promote NSC adhesion and directed neuronal differentiation, and inhibit astroglial scar formation. This significantly improved hindlimb motor coordination and pain perception in injured mice, confirming the excellent carrier potential of gelatin-based, smart-responsive, injectable hydrogels in spinal cord injury repair.

Furthermore, due to the spinal cord’s unique electrical and mechanical properties, the repair of spinal cord injuries via injection of conductive hydrogels has emerged as a promising approach, particularly for irregular or large gaps. For example, Du et al. [155] utilised dopamine-modified gelatin methacrylate (GelMA-DA) as a matrix to construct an injectable conductive hydrogel loaded with Zn@EGCG-MXene. This hydrogel exhibits mechanical modulus and electrical conductivity that closely match those of natural spinal cord tissue, and its shear-thinning properties allow it to flexibly fill irregular injury defects. As shown in Figure 9b, upon implantation, this conductive hydrogel releases Zn@EGCG in response to the high ROS environment, effectively scavenging ROS and inducing M2-type macrophage polarisation. Following combined electrical stimulation, it activates the PI3K/AKT signaling pathway to promote the directed differentiation of endogenous NSCs into neurons. This synergistic effect significantly reduces cystic cavity formation, reconstructs neural circuits, and enhances hindlimb motor coordination in rats, thereby providing a novel strategy for SCI repair that combines electrophysiological regulation with microenvironmental remodeling. Furthermore, excessive ROS at the SCI site can induce neuronal apoptosis and inhibit nerve regeneration; therefore, the development of a hydrogel capable of scavenging ROS is of paramount importance. Lin et al. [156] utilised the dynamic covalent bond between gelatin and sodium alginate to develop a ROS-responsive, injectable hydrogel (GEL-EXO) loaded with exosomes. As shown in Figure 9c, in the high-ROS microenvironment following SCI, this hydrogel responsively releases exosomes and effectively scavenges ROS, thereby promoting macrophage polarisation towards the M2 anti-inflammatory phenotype and achieving dual recovery of motor and bladder autonomic functions. Research indicates that extracellular vesicles (EVs) derived from mesenchymal stem cells (MSCs) exhibit neuroprotective effects, including alleviating neuroinflammation and promoting neurogenesis. Furthermore, EVs derived from three-dimensional (3D) cultured MSCs (3EVs) exhibit significantly superior angiogenic and neurotrophic effects compared to those from two-dimensional (2D) cultures [157]. Based on this, in another study on anti-inflammatory treatment for SCI, Cao et al. [151] employed engineering techniques to enhance the efficacy of EVs by encapsulating dexamethasone (Dxm) within 3EVs to form 3EVs-Dxm, thereby boosting the anti-inflammatory effect. To achieve long-term retention of 3EVs-Dxm within SCI tissue and ROS-responsive release, the research team developed an injectable 3EVs-Dxm-Gel hydrogel. As shown in Figure 9d, after 3EVs-Dxm was modified with 2,3-dihydroxy groups, it cross-linked with phenylboronic acid-modified HA via phenylboronic ester bonds; the remaining PBA then cross-linked with the polyphenolic hydroxyl groups of TA, forming a stable three-dimensional network. Owing to its excellent injectability and mechanical properties, the 3EVs-Dxm-Gel can precisely fill the SCI site. In this context, TA exerts an antioxidant effect, whilst the phenylboronic acid ester bonds enable the responsive release of EVs at the injury site upon ROS stimulation. In a rat model of SCI, 3EVs-Dxm-Gel demonstrated excellent biocompatibility; not only did it suppress oxidative damage and inflammation, but it also promoted nerve fibre regeneration and angiogenesis, ultimately significantly improving the rats’ motor and sensory functions and effectively facilitating SCI repair.

In the field of spinal cord injury (SCI) repair, future developments may involve the use of new manufacturing technologies, such as 3D and 4D printing, to create hydrogels that mimic the layered structure of the natural human spinal cord whilst maintaining good injectability and flexibility, thereby meeting the requirements for filling damaged areas. Concurrently, bioactive hydrogel therapies could be integrated with biosensing, bioimaging and physical diagnostic techniques such as MRI and CT, utilising functional nanoparticles and molecular imaging agents. Furthermore, given that current research is largely concentrated on the preclinical stages of traditional drugs and growth factors, further clinical trials are required to develop safe and effective therapeutic agents, thereby advancing the translation of SCI repair research into clinical applications.

### 4.5. Repair of Nerve Injuries

Peripheral nerve injury (PNI) is a highly challenging neurological condition that affects millions of people worldwide each year [158]. Such injuries are often caused by accidents or sports-related incidents, frequently resulting in severe stretching, compression or crushing of nerve tissue, which poses significant obstacles to the restoration of neurological function in patients [159]. Currently, surgical grafting remains the primary method for treating extensive nerve injuries, with options including autologous nerves, allogeneic nerves, decellularised nerve grafts and bioengineered synthetic conduits. Whilst autologous nerve grafting is widely recognised as the gold standard for surgical repair, it faces numerous challenges in clinical practice, including limited availability of donor tissue, increased surgical trauma, and the potential for neuroma formation [160,161]. Consequently, identifying effective alternatives to autologous nerve grafting has become a key objective for researchers. Among numerous alternatives, nerve guidance conduits (NGCs) made from biodegradable materials have demonstrated significant potential for peripheral nerve repair, owing to their ability to provide structural support and a biomimetic environment. However, traditional NGCs have notable shortcomings; they are unable to adapt to the cyclic and intense mechanical stimuli—such as tension, bending and compression—experienced during peripheral nerve regeneration, making it difficult for them to meet the practical demands of nerve regeneration [162].

**Figure 10:**
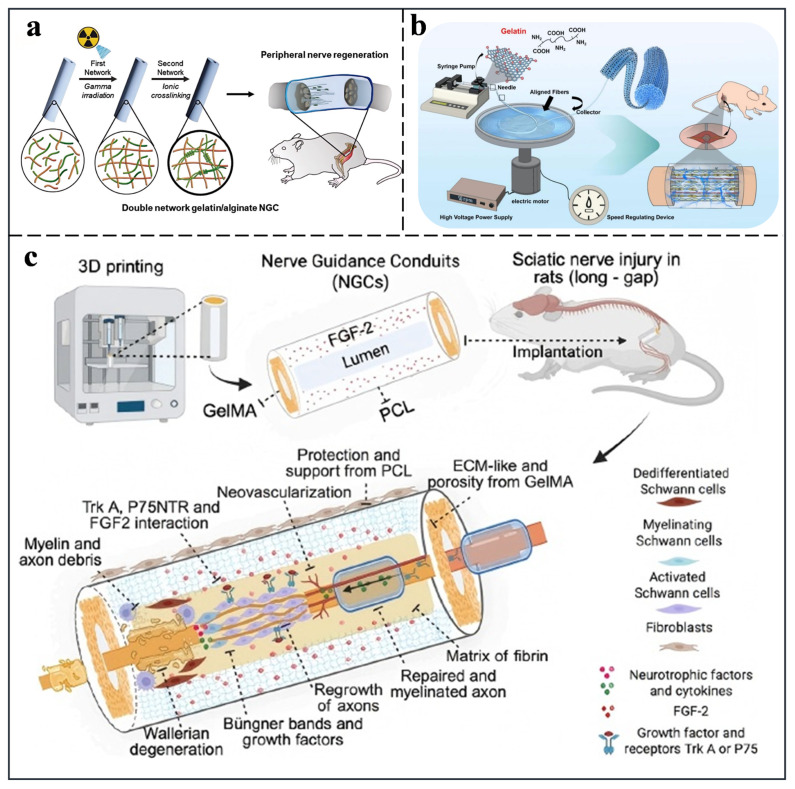
(**a**) Gelatin/alginate dual-network hydrogel nerve conduit for peripheral nerve regeneration [165]. (**b**) Schematic illustration of the alignment of conductive fibres [166]. (**c**) 3D-printed PCL/GelMA bilayer nerve conduit loaded with FGF-2 for promoting nerve regeneration [167].

In recent years, research has shown that ES is an effective method for promoting nerve regeneration [163]. Conductive hydrogels can mimic the electrophysiological environment necessary for the transmission of bioelectric signals, thereby promoting Schwann cell function, axonal regeneration and myelination [164]. Inspired by this, researchers have combined conductive hydrogels with NGCs to develop various conductive hydrogel-based NGCs, with the aim of providing an optimal repair solution for peripheral nerve regeneration. To overcome traditional limitations, as shown in Figure 10a, Kim et al. [165] developed an NGC based on a gelatin/sodium alginate dual-network hydrogel prepared without chemical cross-linking. Its tensile strength (71.4 kPa), elastic modulus (77.0 kPa) and resistance to twisting were significantly superior to those of a gelatin single-network hydrogel, meeting the requirements for surgical procedures and long-term stability in PNI repair. This hydrogel NGC possesses a porous structure resembling the extracellular matrix and good biocompatibility, supporting the adhesion, proliferation, and neurite outgrowth of PC12 cells. In a 10 mm rat sciatic nerve defect model, the sciatic nerve function index reached −64.7 ± 0.6 at 12 weeks post-surgery, significantly outperforming commercial silicone conduits. Multiple research findings collectively confirm that this gelatin-based dual-network hydrogel (NGC) can safely and effectively promote nerve regeneration and motor function recovery by optimising mechanical properties and the microenvironment, demonstrating significant application value in PNI repair. In a similar study, Yang et al. [166] used gelatin and CS as matrix materials to fabricate carbon-based conductive hydrogels doped with CNTs or GO via electrospinning, forming an oriented fibre structure (Figure 10b). The compressive elastic modulus of these hydrogels matches that of neural tissue, enabling them to adapt to the mechanical environment during neural regeneration and thus serve as an ideal candidate material for the repair of peripheral nerve injuries.

Furthermore, to address the limitations of NGCs’ biological activity and their immunocompatibility challenges, the researchers found that integrating stem cells or exosomes with NGCs can significantly enhance the efficacy of neural tube repair (Figure 10c), thereby facilitating their clinical application [167]. Kim et al. [168] encapsulated human umbilical cord mesenchymal stem cells (ucMSCs) within a dual-network gel (NGC) based on gelatin methacrylate and sodium alginate, thereby constructing an active neural conduit loaded with stem cells. This gelatin-based dual-network hydrogel not only possesses excellent mechanical properties, meeting the requirements for surgical suturing and long-term in vivo stability, but also provides a favourable growth microenvironment for ucMSCs, enabling them to maintain high survival rates and exert their effects via paracrine mechanisms. In a 10 mm rat sciatic nerve defect model, this gelatin-based NGC loaded with stem cells significantly downregulated the expression of inflammatory factors (TNF-α, IL-6) and promoted angiogenesis and neurite outgrowth; 12 weeks post-surgery, the sciatic nerve function index reached −72 ± 4, with the positive areas for Schwann cell marker S-100 and neuronal marker NF-200 reaching 51.9% and 64.1% respectively, and with myelin thickness and axon diameter both significantly superior to those of acellular NGC and commercial silicone conduits. Furthermore, it effectively alleviated muscle atrophy and fibrosis, confirming that gelatin hydrogel, when loaded with stem cells, can further enhance the biological activity and immunocompatibility of NGC, providing a novel integrated solution combining mechanical support and biological regulation for PNI repair.

## 5. Conclusions and Outlook

This paper provides a systematic review of the latest advances in gelatin-based multifunctional hydrogels for the repair of sports injuries. From a molecular design perspective, gelatin, owing to its intrinsic biological activity (RGD/MMP sequences) and exceptional modifiability, has become an ideal foundation for the construction of next-generation tissue engineering scaffolds. Through the incorporation of functional modules such as conductivity, antibacterial properties, self-healing capabilities, smart responsiveness and tissue adhesion, novel gelatin hydrogels have evolved from simple filling materials into intelligent therapeutic platforms capable of actively regulating the immune microenvironment, restoring electrophysiological signals and adapting to dynamic mechanical loads. They demonstrate immense clinical potential in osteochondral regeneration, tendon/ligament repair and neural function reconstruction.

Looking forward, the paradigm of sports injury repair will be profoundly reshaped by the convergence of multifunctional gelatin hydrogels with cutting-edge emerging technologies. Specifically, artificial intelligence (AI)-assisted biomaterial design will enable high-throughput screening and precise prediction of hydrogel properties, significantly accelerating the customized development of patient-specific scaffolds. Furthermore, 4D bioprinting technology introduces “time” as the fourth dimension, allowing smart hydrogels to undergo dynamic shape or functional transformations in response to physiological stimuli, thereby achieving unprecedented spatiotemporal matching with the dynamic tissue remodeling process. From an electrophysiological perspective, the integration of advanced bioelectronic interfaces into hydrogel networks will facilitate closed-loop monitoring and on-demand stimulation, enabling real-time assessment and intervention for neuromuscular injuries. In terms of biological functionalization, exosome-loaded hydrogels can leverage cell-free therapy to effectively modulate the local immune microenvironment and promote angiogenesis with minimized immunogenicity. Meanwhile, CRISPR-engineered cell–hydrogel systems offer a revolutionary approach by precisely correcting local gene expression to overcome intrinsic healing deficiencies. Ultimately, the development of smart rehabilitation-integrated biomaterials—which seamlessly couple hydrogel scaffolds with wearable biosensors—will create a comprehensive “diagnosis–repair–rehabilitation” closed-loop system. These interdisciplinary advancements will undoubtedly push the boundaries of regenerative sports medicine, transforming passive implants into active, intelligent therapeutic platforms [169,170].

However, the field still faces numerous significant challenges in bridging the gap between laboratory research and clinical translation, which also points the way for future research:

(1) Decoding long-term in vivo degradation and immunomodulation: The majority of current studies are restricted to short-term, small-animal models, which insufficiently replicate human sports injuries. There is a critical need to systematically track the long-term metabolic fate of hybrid degradation products (especially non-degradable nanomaterials) and evaluate their chronic systemic immunotoxicity. Furthermore, precisely synchronizing the scaffold’s biodegradation kinetics with the specific regenerative timelines of varying tissues—such as avascular cartilage versus highly vascularized muscle—remains a formidable and unresolved challenge.

(2) Balancing mechanical properties and biological functionality: When striving for high strength and toughness to withstand mechanical loads (e.g., in dual-network and nanocomposite materials), this often comes at the expense of the material’s porosity and biocompatibility. In the future, we need to explore ‘decoupling’ design strategies or utilise 4D printing technology to construct biomimetic scaffolds with anisotropic, gradient mechanical structures to perfectly replicate the mechanical microenvironment of natural tissues.

(3) Synergistic effects of multifunctional integration: Current research often endows materials with multiple functions through simple ‘addition’, but interference may occur between the various functional modules. Future research should focus on elucidating the synergistic biological mechanisms underlying different physical and chemical signals (such as electrical stimulation, drug delivery and mechanical support) in tissue regeneration, and utilise high-throughput screening and AI-assisted design to optimise material formulations, thereby achieving a therapeutic effect where ‘1 + 1 > 2’.

(4) GMP compliance, regulatory approval, and commercialization barriers: The clinical translation of GBMHs is heavily bottlenecked by regulatory considerations. The complex chemical compositions of multi-functional and smart-responsive networks complicates quality control, posing immense challenges for standardization. To transition from bench to bedside, future research must move away from complex laboratory synthesis toward robust, GMP-compliant fabrication protocols. Validating cost-effective, scalable production lines, securing batch consistency, and ensuring zero performance loss after clinically acceptable sterilization are the absolute pre-requisites for navigating the regulatory approval process and achieving successful market commercialization.

(5) Cross-disciplinary integration with agricultural science: The sustainable development of gelatin-based hydrogels relies on agricultural science and technology. Livestock and aquaculture by-products are the main raw materials of gelatin, and green agricultural processing technologies enable their high-value utilization. Natural active components for hydrogel functionalization are all derived from agricultural products, and standardized agricultural quality control ensures their batch consistency, supporting large-scale production and clinical translation.

In summary, with the deepening integration of materials science, manufacturing technology, and regenerative medicine, there is good reason to believe that functionalised gelatin hydrogels will play a central role in the future of precision sports medicine, offering patients more effective, minimally invasive treatment options.

## Figures and Tables

**Table 1:** Comparison of gelatin and alternative foundational biomaterials.

Matrix	Mechanical Adaptability	Biodegradation Kinetics	Cell Affinity	Translational Bottlenecks	Refs.
Gelatin and GelMA	High viscoelasticity and fatigue resistance	MMP-responsive; syncs with tissue remodeling	Excellent (inherent RGD sequences)	Batch variability; sterilization degradation	[7,8]
Alginate	Brittle; poor cyclic elastic recovery	Unpredictable ion-exchange; prone to early collapse	Poor (requires RGD grafting)	No native cell targets; potential immune bursts	[35]
Collagen	Flexible but mechanically weak under high stress	Rapid enzymatic hydrolysis; hard to tune	Excellent (native ECM signaling)	High cost; zoonotic disease risks	[36]
Hyaluronic Acid (HA)	High lubricity; low initial shear modulus	Rapid enzymatic cleavage by hyaluronidases	Good (CD44 receptor binding)	High swelling causing localized tissue compression	[37]
Polyethylene Glycol (PEG)	Tunable elasticity; lacks viscoelastic dissipation	Non-degradable or slow hydrolytic cleavage	Inert (hinders cell infiltration)	In vivo accumulation of non-degradable fragments	[38]
Synthetic Polymers (PLA and PCL)	High tensile strength and initial load-bearing	Slow bulk degradation; acidic byproducts	Poor (highly hydrophobic)	Mechanical mismatch; chronic FBR	[28]

**Table 2:** Functional modules and sports injury applications of gelatin hydrogels.

Platform Type	Additives and Crosslinkers	Target Properties	Sports Injury Applications	Engineering Advantages	Limitations	Refs.
Conductive	CNTs, MXene, ionic liquids, metal salts	High conductivity (0.01–0.93 S/m)	Monitors joint kinematics; electro-stimulated muscle or nerve repair	High signal-to-noise ratio	Nanofiller agglomeration; free ion leakage	[45,46,50,54,55]
Antimicrobial	Chitosan, silver nanoparticles, antibiotics	Sustained sterilization	Treats severe turf abrasions; prevents ACL implant infections	Broad-spectrum; avoids systemic toxicity	Burst release risks; potential cytotoxicity	[65,66,68,69,73]
Self-healing	Dynamic bonds, host–guest complexes, Schiff bases	Autonomous room-temperature repair	Withstands cyclic joint bending; supports torn tendon repair	Instant trigger-free interfacial healing	Low initial modulus; slow healing kinetics	[91,92,94,95]
Adhesive	Schiff bases, multiple hydrogen bonds	Strong wet adhesion (up to 48.67 kPa)	Hemostasis in acute sports trauma; bonds wet moving tissues	Rapid formation; sutureless sealing	Cohesion-adhesion trade-off; aldehyde toxicity	[121]
Responsive	ROS-responsive linkages, carbene crosslinkers	Stimuli-triggered drug delivery	Reverses post-traumatic osteoarthritis; repairs focal cartilage defects	Adapts to local inflammatory microenvironment	Complex synthesis; potential clearance issues	[115,116]

## Data Availability

Not applicable.
